# Systematic analysis of snRNA genes reveals frequent *RNU2-2* variants in dominant and recessive developmental and epileptic encephalopathies

**DOI:** 10.1038/s41588-026-02547-5

**Published:** 2026-03-30

**Authors:** Elsa Leitão, Amandine Santini, Benjamin Cogne, Miriam Essid, Maria Athanasiadou, Christy W. LaFlamme, Pierre Marijon, Virginie Bernard, Kevin Jousselin, Nicolas Chatron, Giulia Barcia, Boris Keren, Cyril Mignot, Perrine Charles, Thomas Besnard, Robin Paluch, Jean-Madeleine de Sainte Agathe, Edith P. Almanza Fuerte, Soham Sengupta, Mathieu Milh, Francis Ramond, Talia Allan, Isabelle An, Camila Araujo, Stéphanie Arpin, Christina Austin-Tse, Stéphane Auvin, Sarah Baer, Nadia Bahi-Buisson, Mads Bak, Magalie Barth, Stéphanie Baulac, Nathalie Bednarek-Weirauch, Matthias Begemann, Mark F. Bennett, Uriel Bensabath, Stéphane Bézieau, Rakia Bhouri, Margaux Biehler, Trine Bjørg Hammer, Julie Bogoin, Emilie Bonanno, Simon Boussion, Céline Bris, Adelaide Brosseau-Beauvir, Ange-Line Bruel, Audrey Briand-Suleau, Julien Buratti, Tristan Celse, Pascal Chambon, Nicole Chemaly, Bertrand Chesneau, Estelle Colin, Maxime Colmard, Cindy Colson, Solène Conrad, Thomas Courtin, Isabelle Creveaux, Anne-Charlotte Cullier, Louis T. Dang, Anne de Saint Martin, Caroline de Vanssay de Blavous Legendre, Bénédicte Demeer, Anne-Sophie Denommé-Pichon, Philine Diekhoff, Stephanie DiTroia, Martine Doco-Fenzy, Christèle Dubourg, Charlotte Dubucs, Stéphanie Ducreux, Louis Dufour, Romain Duquet, Benjamin Durand, Salima El Chehadeh, Miriam Elbracht, Laurence Faivre, Marie Faoucher, Anne Faudet, Sylvie Forlani, Mélanie Fradin, Pauline Gaignard, Benjamin Ganne, Aurore Garde, Justine Géraud, Deepak Gill, Alice Goldenberg, David Grabli, Coraline Grisel, Sophie Gueden, Paul Gueguen, Anne-Marie Guerrot, Agnès Guichet, Tobias B. Haack, Nina Härting, Martin Georg Häusler, Solveig Heide, Theresia Herget, Bénédicte Héron, Delphine Héron, Johanna Herwig, Mathilde Heulin, Tess Holling, Clara Houdayer, Bertrand Isidor, Aurélia Jacquette, Louis Januel, Nolwenn Jean-Marçais, Frank J. Kaiser, Sabine Kaya, Chontelle King, Marina Konyukh, Florian Kraft, Jeremias Krause, Rémi Kirstetter, Alma Kuechler, Ingo Kurth, Kerstin Kutsche, Audrey Labalme, Jean-Serene Laloy, Vincent Laugel, Floriane Le Bricquir, Anne-Sophie Lèbre, Marine Lebrun, Eric Leguern, Jonathan Levy, Nico Lieffering, Stanislas Lyonnet, Kevin Lüthy, Sian M. W. Macdonald, Lamisse Mansour-Hendili, Julien Maraval, Iris Marquardt, Carolin Mattausch, Sandra Mercier, Olfa Messaoud, Godelieve Morel, Jérémie Mortreux, Arnold Munnich, Rima Nabbout, Sophie Nambot, Vincent Navarro, Ashana Neale, Laetitia Nguyen, Mathilde Nizon, Frédérique Nowak, Melanie C. O’Leary, Sylvie Odent, Naomi Meave Ojeda, Valérie Olin, Simone Olivieri, Katrin Õunap, Lynn S. Pais, Eleni Panagiotakaki, Olivier Patat, Laurence Perrin-Sabourin, Florence Petit, Christophe Philippe, Amélie Piton, Marc Planes, Céline Poirsier, Antoine Pouzet, Clément Prouteau, Sylvia Quéméner-Redon, Mathilde Renaud, Anne-Claire Richard, Marlène Rio, Clotilde Rivier, Florence Robin-Renaldo, Paul Rollier, Massimiliano Rossi, Agathe Roubertie, Valentin Ruault, Maïlys Rupin-Mas, Pascale Saugier-Veber, Aline Saunier, Russell Saneto, Elisabeth Sarrazin, Catherine Sarret, Elise Schaefer, Caroline Schluth-Bolard, Amy Schneider, Isabell Schumann, Vladimir B. Seplyarskiy, Stephanie Spranger, Thomas Smol, Marc Sturm, Shamil R. Sunyaev, Brian Sperelakis-Beedham, Sarah L. Stenton, Friedrich Stock, Mylène Tharreau, Deniz Torun, Joseph Toulouse, Harshini Thiyagarajah, Stéphanie Valence, Sophie Valleix, Julien Van-Gils, Laurent Villard, Dorothée Ville, Nathalie Villeneuve, Antonio Vitobello, Aurélie Waernessyckle, Jan Wagner, Yvonne Weber, Dagmar Wieczorek, Tom Witkowski, Manya Yadavilli, Tony Yammine, Khaoula Zaafrane-Khachnaoui, Maha S. Zaki, Alban Ziegler, Nuria C. Bramswig, Alban Lermine, Gael Nicolas, Joseph G. Gleeson, Lynette G. Sadleir, Michael S. Hildebrand, Ingrid E. Scheffer, Nicola Whiffin, Anne O’Donnell-Luria, Heather C. Mefford, Pierre Blanc, Julien Thevenon, Camille Charbonnier, Clément Charenton, Christel Depienne, Gaetan Lesca, Caroline Nava

**Affiliations:** 1 Institute of Human Genetics, University Hospital Essen, University Duisburg-Essen, Essen, Germany; 2 Université Rouen Normandie, Normandie Université, Inserm U1245 and CHU Rouen, Department of Genetics and Reference Center for Developmental Abnormalities, Rouen, France; 3 Nantes Université, CHU de Nantes, Service de Génétique Médicale, Nantes, France; 4 Nantes Université, CHU de Nantes, CNRS, INSERM, l’Institut du Thorax, Nantes, France; 5 Laboratoire SeqOIA, Paris, France; 6 Genetics Department, Hospices Civils de Lyon, Lyon, France; 7 Pathophysiology and Genetics of Neuron and Muscle (PNMG), UCBL, CNRS UMR5261 - INSERM, Lyon, France; 8 GCS AURAGEN, Lyon, France; 9 CNRS, Inserm, Université de Strasbourg, IGBMC UMR 7104- UMR-S 1258, Illkirch, France; 10 Department of Integrated Structural Biology, IGBMC, Illkirch, France; 11 Center for Pediatric Neurological Disease Research, St. Jude Children’s Research Hospital, Memphis, TN USA; 12 Graduate School of Biomedical Sciences, St. Jude Children’s Research Hospital, Memphis, Memphis, TN USA; 13 Assistance Publique - Hôpitaux de Paris (APHP), Service de Médecine Génomique des Maladies Rares, Hôpital Necker-Enfants malades, Paris, France; 14 Université Paris Cité, INSERM, IHU Imagine – Institut des maladies génétiques, Paris, France; 15 Département de Génétique Médicale, Assistance Publique - Hôpitaux de Paris (APHP) Sorbonne Université, Hôpital Pitié-Salpêtrière, Paris, France; 16 Centre de Référence Déficiences Intellectuelles de Causes Rares, Paris, France; 17 Service de Neurologie Pediatrique, AP-HM, Marseille, France; 18 Aix Marseille Université, Inserm, INMED, Marseille, France; 19 Département de Génétique, Centre Hospitalier Universitaire de Saint-Etienne, Saint-Etienne, France; 20 Department of Medicine, Epilepsy Research Centre, University of Melbourne, Austin Health, Heidelberg, Victoria Australia; 21 Département de Neurologie, Assistance Publique - Hôpitaux de Paris (APHP) Sorbonne Université, Center of Reference for Rare Epilepsies, ERN EPICARE, Hôpital Pitié-Salpêtrière, Paris, France; 22 Department of Surgery and Anatomy, Ribeirão Preto Medical School, University of São Paulo, Ribeirao Preto, Brazil; 23 Service de Génétique, CHU de Tours, Tours, France; 24 Université de Tours, INSERM, Imaging Brain and Neuropsychiatry iBraiN U1253, Tours, France; 25 Broad Center for Mendelian Genomics, Program in Medical and Population Genetics, Broad Institute of MIT and Harvard, Cambridge, MA USA; 26 Département de Neuropédiatrie, Assistance Publique - Hôpitaux de Paris (APHP), Hôpital Robert-Debré, Paris, France; 27 Université Paris Cité, INSERM NeuroDiderot, Paris, France; 28 Service de Neuropédiatrie, Hôpitaux Universitaires de Strasbourg, Strasbourg, France; 29 Department of Clinical Genetics, Copenhagen University Hospital, Rigshospitalet, Denmark; 30 Department of Medical Genetics, Angers University Hospital, Angers, France; 31 Institut du Cerveau - Paris Brain Institute - ICM, Sorbonne Université, Inserm, CNRS, APHP, Hôpital de la Pitié Salpêtrière, Paris, France; 32 Service de Pédiatrie, CHU Reims, Reims, France; 33 Université Reims Champagne Ardenne (URCA), UFR médecine, Reims, France; 34 CReSTIC/EA 3804, URCA, Reims, France; 35 Center for Human Genetics and Genomic Medicine, Medical Faculty, RWTH Aachen University Hospital, Aachen, Germany; 36 Genetics and Gene Regulation Division, Walter and Eliza Hall Institute of Medical Research, Parkville, Victoria Australia; 37 Department of Medical Biology, The University of Melbourne, Parkville, Victoria Australia; 38 Service de Génétique Médicale, Centre Hospitalier Intercommunal de Créteil (CHIC), Créteil, France; 39 Laboratoire de Diagnostic Génétique, Nouvel Hôpital Civil, Hôpitaux Universitaires de Strasbourg, Strasbourg, France; 40 Danish Epilepsy center, Dianalund, Denmark; 41 CHU Lille, Université Lille, ULR7364 – RADEME, Lille, France; 42 MitoLab, Unité MITOVASC, UMR CNRS 6015, INSERM U1083, SFR ICAT, University Hospital of Angers, Angers, France; 43 Center for Intellectual Disability Reference, Brest University Hospital, Brest, France; 44 Laboratoire de Génomique Médicale, Centre Neomics, FHU-TRANSLAD, Centre de Recherche Translationnelle en Médecine Moléculaire – Inserm UMR1231 équipe GAD, Université Bourgogne Europe, CHU Dijon Bourgogne, Dijon, France; 45 Service de Génétique, Génomique et Procréation, CHU Grenoble Alpes, Grenoble, France; 46 Department of Pediatric Neurology, Reference Center for Rare Epilepsies, Necker Enfants Malades Hospital, Paris, France; 47 Service de Génétique Médicale, CHU Purpan, Toulouse, France; 48 Service de Neuropédiatrie, CHU Montpellier, Montpellier, France; 49 Laboratoire de Génétique Moléculaire, CHU de Clermont-Ferrand, Clermont-Ferrand, France; 50 Service de Pediatrie, CHR Metz-Thionville, Hôpital Mercy, Metz, France; 51 Department of Pediatrics, Michigan Medicine, University of Michigan, Ann Arbor, MI USA; 52 Service de Pédiatrie, Consultation de Neurologie Pédiatrique GHH Jacques Monod, Le Havre, France; 53 Service de Génétique Clinique et Oncogénétique, CLAD Nord-ouest, CHU Amiens-Picardie, Amiens, France; 54 CHIMERE - INSERM UA-21, Université Picardie Jules Verne, Amiens, France; 55 Institute of Human Genetics, University Medical Center Hamburg-Eppendorf, Hamburg, Germany; 56 UF de Génétique Clinique, CHU de Reims, Reims, France; 57 Laboratoire de Génétique Moléculaire et Génomique, FHU GenOMedS, CHU Rennes, Rennes, France; 58 IGDR (Institut de Génétique et Développement de Rennes)-UMR 6290, Université Rennes, CNRS, INSERM, Rennes, France; 59 Département de Pathologie, Institut Universitaire du Cancer Toulouse - Oncopole, Toulouse, France; 60 Service de Génétique Médicale, Institut de Génétique Médicale d’Alsace (IGMA), CHU Strasbourg, Strasbourg, France; 61 Centre de Référence Anomalies du Développement et Syndromes Malformatifs, Université Bourgogne Europe, CHU Dijon Bourgogne, Inserm, CTM UMR1231, équipe GAD, FHU TRANSLAD, Centre de génétique, Centre de référence Déficiences Intellectuelles de Causes Rares et Centre de référence GénoPsy, Dijon, France; 62 Service de Génétique Clinique, Centre de Référence ‘Anomalies du Développement et Syndromes Malformatifs’ de l’Inter-région Ouest, FHU GenOMedS, CHU Rennes Hôpital Sud, Rennes, France; 63 Laboratoire de Biochimie Site Bicêtre, Faculté de Pharmacie, Hôpitaux Universitaires Paris-Saclay, Centre de référence des Maladies Mitochondriales, Filière Filnemu, Paris, France; 64 Laboratoire de Génétique Chromosomique, CHU de Montpellier, Montpellier, France; 65 Centre de Génétique, Université Bourgogne Europe, CHU Dijon Bourgogne, Centre de Référence maladies rares ‘Anomalies du Développement et syndromes malformatifs,’ FHU-TRANSLAD, Dijon, France; 66 Neuropediatric Department, University Hospital Centre Toulouse, Toulouse, France; 67 Kids Neuroscience Centre, Kids Research Institute, Sydney, New South Wales Australia; 68 TY Nelson Department of Neurology and Neurosurgery, The Children’s Hospital at Westmead, Sydney Children’s Hospitals Network, Sydney, New South Wales Australia; 69 Specialty of Child and Adolescent Health, University of Sydney, Sydney, New South Wales Australia; 70 Service de Pédiatrie, Centre Hospitalier Intercommunal de Créteil, Créteil, France; 71 Department of Pediatric Neurology, Angers University Hospital, Angers, France; 72 Institute of Medical Genetics and Applied Genomics, University of Tübingen, Tübingen, Germany; 73 Department of Pediatrics, University Hospital, Division of Neuropediatrics and Social Pediatrics, Rheinisch-Westfälische Technische Hochschule Aachen, Aachen, Germany; 74 Département de Neurologie Pédiatrique, Assistance Publique - Hôpitaux de Paris (APHP) Sorbonne Université, Fédération Hospitalo-Universitaire I2-D2, Hôpital Armand Trousseau-La Roche Guyon, Paris, France; 75 Service de Neuropédiatrie, Hôpital Jean-Verdier, Bondy, France; 76 Consultation de génétique, CCMR ANDDI rare, Centre Hospitalier d’Alençon, Alençon, France; 77 Department of Paediatrics and Child Health, University of Otago, Wellington, New Zealand; 78 Département de Génétique Médicale, Hôpital Henri Mondor, Assistance Publique des Hôpitaux de Paris, Créteil, France; 79 Service de Pédiatrie, Nantes Université, CHU de Nantes, Service de Pédiatrie, Nantes, France; 80 Laboratoire de Génétique, CHU de Reims, Reims, France; 81 INSERM U1266 (Krebs team), Institute of Psychiatry and Neuroscience of Paris (IPNP),Université Paris Cité, Paris, France; 82 Département de génétique, Assistance Publique - Hôpitaux de Paris (APHP), Hôpital Robert-Debré, Paris, France; 83 MVZ Klinikum Oldenburg, Oldenburg, Germany; 84 Harvard Medical School, Boston, MA USA; 85 Division of Genetics and Genomics, Boston Children’s Hospital, Harvard Medical School, Boston, MA USA; 86 Center for Genomic Medicine, Massachusetts General Hospital, Harvard Medical School, Boston, MA USA; 87 Service de Génétique, CHU (Centre Hospitalier Universitaire) de La Réunion, Saint-Denis, France; 88 Health Technologies Institute, Inserm, Paris, France; 89 Department of Neurosciences, University of California San Diego, La Jolla, CA USA; 90 Rady Children’s Institute for Genomic Medicine, San Diego, CA USA; 91 Department of Genetics and Personalized Medicine, Institute of Clinical Medicine, University of Tartu, Tartu, Estonia; 92 Department of Clinical Genetics, Genetics and Personalized Medicine Clinic, Tartu University Hospital, Tartu, Estonia; 93 Department of Clinical Epileptology, Sleep Disorders and Functional Neurology in Children, University Hospital of Lyon (HCL), Lyon, France; 94 Lyon Neuroscience Research Center, Inserm U1028/CNRS UMR 5292, Lyon, France; 95 Laboratoire de Génétique Médicale, CHR Metz-Thionville, Hôpital Mercy, Metz, France; 96 Institute of Genetics and Cellular and Molecular Biology (IGBMC), INSERM-U964, CNRS-UMR7104, University of Strasbourg, Illkirch, France; 97 Medical Genetics Department, Brest University Hospital, Brest, France; 98 University of Brest, Inserm, EFS, UMR 1078, GGB, Brest, France; 99 Service de Génétique Clinique, CHRU Nancy, Vandoeuvre les Nancy, France; 100 Department of Pediatrics, Hôpital Nord-Ouest, Villefranche sur Saône, France; 101 INM, INSERM U 1298, Montpellier, France; 102 Clinical Genetic Unit, Reference Center for Congenital Anomalies, CHU Montpellier, University of Montpellier, Montpellier, France; 103 Division of Pediatric Neurology, Neuroscience Institute, Norcliff Center for Integrative Brain Research, Seattle Children’s Hospital/University of Washington, Seattle, WA USA; 104 Caribbean Reference Center for Neuromuscular Diseases, University Hospital Fort de France, Martinique, France; 105 Neurologie Pédiatrique et Génétique Médicale, CHU de Clermont-Ferrand, Clermont-Ferrand, France; 106 UMRS 1112, INSERM, Université de Strasbourg, Strasbourg, France; 107 Department of Medical Genetics, Centre of Medical Genetics, University and University Hospital Münster, Münster, Germany; 108 Institute of Human Genetics, Leipzig University Medical Center, Leipzig, Germany; 109 Department of Biomedical Informatics, Harvard Medical School, Boston, MA USA; 110 Division of Genetics, Brigham and Women’s Hospital, Harvard Medical School, Boston, MA USA; 111 MVZ Humangenetik Bremen, Limbach Genetics, Bremen, Germany; 112 Laboratory of Molecular Genetics of Rare Diseases, Montpellier University Hospital, Montpellier, France; 113 Department of Medical Genetics, Gulhane Faculty of Medicine, University of Health Sciences, Ankara, Turkey; 114 Department of Systemic and Organ Diseases, Assistance Publique - Hôpitaux de Paris (APHP), Paris City University, Genomic Medicine, Cochin Hospital, Paris, France; 115 Service de Génétique Médicale, Centre Hospitalier Universitaire (CHU) de Bordeaux, Bordeaux, France; 116 INSERM U1211, University of Bordeaux, Bordeaux, France; 117 Service de Génétique Médicale, AP-HM, Marseille, France; 118 Inserm, MMG, U1251, Aix Marseille Université, Marseille, France; 119 Department of Pediatric Neurology and Reference Center for Rare Children Epilepsy and Tuberous Sclerosis, Hôpital Femme Mere Enfant, Centre Hospitalier Universitaire de Lyon, Lyon, France; 120 Department of Neurology, Epilepsy Center Ulm, University Hospital Ulm, Ulm, Germany; 121 Department Neurology, Section of Epileptology, Medical Faculty, University RWTH Aachen, Aachen, Germany; 122 Institute of Human Genetics, Medical Faculty and University Hospital Düsseldorf, Heinrich Heine University Düsseldorf, Düsseldorf, Germany; 123 Centre Hospitalier Universitaire de Nice, Inserm U1081, CNRS UMR7284, IRCAN, Université Côte d’Azur, Nice, France; 124 Clinical Genetics Department, Human Genetics and Genome Research Institute, National Research Centre, Cairo, Egypt; 125 Neuroscience Group, Murdoch Children’s Research Institute, Parkville, Victoria Australia; 126 Department of Paediatrics, University of Melbourne, Royal Children’s Hospital, Melbourne, Victoria Australia; 127 Florey Institute and Murdoch Children’s Research Institute, Melbourne, Victoria Australia; 128 Big Data Institute, University of Oxford, Oxford, UK; 129 Centre for Human Genetics, University of Oxford, Oxford, UK; 130 Institut for Advanced Biosciences, Université Grenoble Alpes, INSERM U 1209, CNRS UMR 5309, Grenoble, France; 131 Department of Biostatistics and Reference Center for Developmental Abnormalities, Université Rouen Normandie, Normandie Université, Inserm U1245 and CHU Rouen, Rouen, France

**Keywords:** Neurodevelopmental disorders, Genetics research, Transcriptomics, Epilepsy

## Abstract

Small nuclear RNAs (snRNAs) are essential components of the spliceosome. De novo variants in snRNA genes *RNU4-2* (ReNU syndrome), *RNU5B-1* and *RNU2-2* have been linked to dominant neurodevelopmental disorders (NDDs), revealing a large unexpected contribution of noncoding RNA genes to genetic diseases. Here, through international collaborations, we analyze systematically 200 potentially functional snRNA genes in a French cohort of 34,329 people with rare disorders. We report *RNU2-2* variants in 141 individuals, including 35 with recurrent dominant pathogenic variants and 91 affected members from 73 families with biallelic variants. Recessive *RNU2-2* NDD is at least twice as frequent as the dominant form and often involves a de novo variant in trans with an inherited allele, consistent with the high mutability of snRNA genes. Dominant and recessive *RNU2-2* NDDs share overlapping clinical features, with frequent epilepsy. Blood transcriptomics and DNA methylation analyses revealed subtle, variant-specific effects on splicing and episignatures. Our results support a gradient-of-impact model bridging dominant and recessive inheritance, and establish *RNU2-2* variants as a principal contributor to NDDs, nearly as prevalent as ReNU syndrome.

## Abstract (web summary)

Analyses of snRNA genes in a French cohort of people with rare disorders, with validation through international collaboration, identify monoallelic and biallelic variants in *RNU2-2* as frequent causes of neurodevelopmental disorders with epilepsy.

## Main

Small nuclear RNAs (snRNAs) are noncoding RNAs essential for RNA processing and splicing of premessenger RNAs (mRNA). The spliceosome—a dynamic ribonucleoprotein (RNP) complex that catalyzes splicing—depends on five uridine-rich snRNAs for its assembly and function. In mammals, two spliceosome types operate according to intron class: the major spliceosome excises >99% of introns with GU–AG splice sites (U2-type) using snRNAs U1, U2, U4, U5 and U6, whereas the minor spliceosome removes rare introns with AU–AC or GU–AG splice sites (U12-type) with snRNAs U11, U12, U4atac and U6atac, sharing U5 with the major complex^1–3^.

Although minor spliceosome snRNA genes are single-copy, major spliceosome genes have several functional copies^4^. Human genomes contain at least two U4, five U5 and five U6 functional genes. U1 is expressed from at least four identical copies (*RNU1-1* to *RNU1-4*) on chromosome (chr) 1p36.13, plus more than 30 variant U1 (*RNVU1*) genes on 1q21.1–q23.3^5–7^. Most U2 genes (‘*RNU2-1’*) are organized in large tandem arrays on chr17q21.31, with copy numbers ranging from 6 to >80 per chromosome^8^.

Biallelic pathogenic variants were first described in *RNU4ATAC* and cause phenotypically variable developmental disorders: microcephalic osteodysplastic primordial dwarfism type I, Taybi–Linder, Lowry–Wood or Roifman syndromes^9–13^. Recessive variants in *RNU12* may lead to craniosynostosis, anal anomalies and skin lesions and/or spinocerebellar ataxia^14,15^ although definitive evidence is missing.

In 2024, de novo variants in the major spliceosome gene *RNU4-2* were shown to cause ReNU syndrome (OMIM 620851)—one of the most common known NDDs^16,17^. These dominant variants are located within an 18-bp critical region spanning the T loop and part of stem III, facing the U6 ACAGAGA box that enables 5′ splice site (5′SS) recognition^16,18^. *RNU4-2* pathogenic variants are associated with mild, but specific, widespread splicing and methylation abnormalities in blood cells of affected people, the degree of which correlates with disease severity^16,18^. De novo variants in *RNU5B-1* (and possibly *RNU5A-1*) clustering in the U5 5′ loop I lead to NDD with variable malformations^18,19^. The recent discoveries of biallelic variants in other regions of *RNU4-2* in NDD patients and heterozygous variants in *RNU4-2* (and genes expressing U6) in families with retinitis pigmentosa^20^ has expanded its mutational spectrum and added complexity to variant interpretation^21,22^.

Identifying variants in snRNA genes is complicated by sequence redundancy and incomplete annotations, especially when distinguishing functional genes from pseudogenes. De novo pathogenic variants in *RNU2-2P*, initially annotated as a pseudogene, were linked recently to a dominant NDD with epilepsy^19,23^. Because *RNU2-2P* is expressed at levels similar to *RNU2-1*, it was reclassified as *RNU2-2*^19,23,24^. However, unlike *RNU4-2*, no splicing anomalies were detected in blood transcriptomes of patients with *RNU2-2* variants^23^. Strikingly, active snRNA (and tRNA) genes are hypermutable, with up to tenfold more de novo variants than other genomic regions—a characteristic that may help distinguish functional genes from pseudogenes^25^.

In this study, we build on our previous work encompassing 50 Human Genome Organization (HUGO) Gene Nomenclature Committee (HGNC)-approved snRNA genes^18^, extending it to investigate variants in possibly functional snRNA genes systematically in a large French cohort with rare diseases. This led us to report a highly prevalent recessive NDD with epilepsy caused by biallelic variants in *RNU2-2* that was validated through international collaborations.

## Results

### Identification of potentially functional spliceosomal snRNA genes

**Fig. 1:**
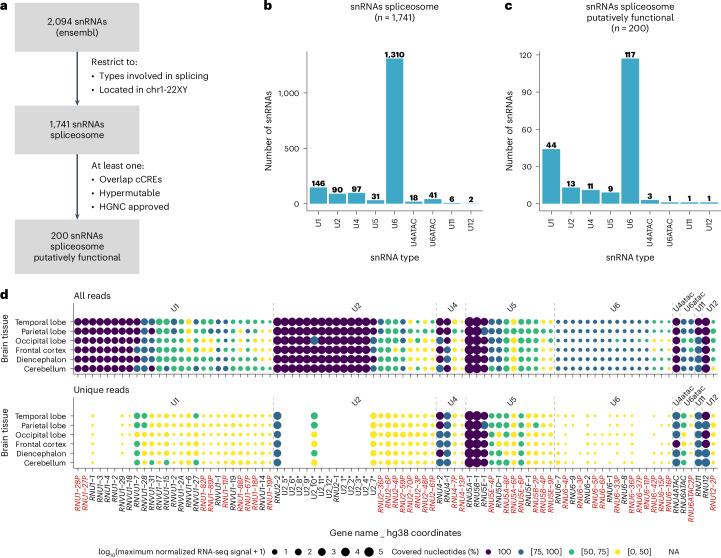
Systematic in silico analysis of possible functional snRNAs. **a**, Filtering strategy used to retain genes expressing possible functional snRNAs. **b**,**c**, Number and distribution of annotated spliceosomal snRNAs before (**b**) and after (**c**) filtering. **d**, Expression of snRNAs in the human brain from ENCODE small RNA-seq data. Top: all mapped reads (including multimapped). Bottom: uniquely mapped reads. Expression is shown as log_10_ of the maximum normalized RNA-seq signal. Colors indicate the proportion of the snRNA length covered by mapped reads: 100% (purple), 75–99% (dark blue), 50–74% (green), <50% (yellow). Genes in red correspond to annotated pseudogenes. Asterisk, genes expressing U2-1 copies within the chr1 cluster lack HGNC approved symbols and were numbered in ascending order of their genomic coordinates. NA, not applicable.

To distinguish functional snRNA genes from pseudogenes, we performed an in silico analysis of all annotated snRNA genes (Methods). The Ensembl database contains 2,094 snRNA genes in the hg38 reference genome: 1,741 are spliceosomal snRNA genes, and the remainder have other functions or are not on identifiable chromosomes. We prioritized genes overlapping proximal *cis*-regulatory elements (cCREs; from ENCODE^26^), reported as hypermutable^25^, or HGNC-approved^18^. This analysis yielded 200 potentially functional snRNA genes, including 147 ‘pseudogenes’ and *RNU2-2* (Fig. 1a and Supplementary Table 1). The breakdown of snRNA types aligns with previous observations: minor spliceosome snRNAs exist mainly as single copies (range 1–3), whereas major spliceosome snRNAs are present in many more copies (range 11–117). U6 has the highest number of copies and U5, shared by both complexes, is intermediate (*n* = 9; Fig. 1b,c).

In parallel, we reanalyzed an ENCODE brain small RNA sequencing (RNA-seq) dataset^27^. We performed two analyses: one using all mapped reads, including multimapped, and another restricted to uniquely mapped reads. The first captures expression from all identical copies, whereas the second reflects only uniquely assignable snRNAs. In total, 87 putatively functional snRNAs, including 39 annotated as pseudogenes, were detectable in the human brain (Fig. 1d), raising the possibility that more NDDs are driven by snRNA variants.

### Analysis of de novo and biallelic variants in putatively functional snRNA genes in the Plan France Médecine Génomique 2025 cohort

**Extended Data Fig. 1:**
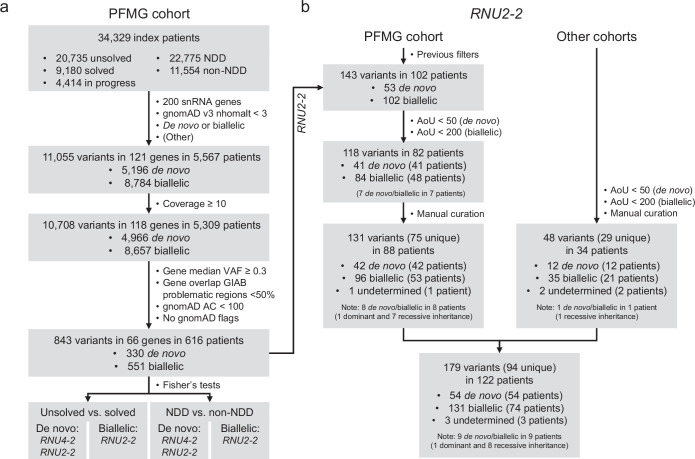
Details of the PFMG cohort and variant filtering strategy. **a**, Number of patients and *de novo* or biallelic variants in snRNA genes the PFMG cohort. Supplementary Fig. 4 shows the same data in the SeqOIA and Auragen subcohorts separately. **b**, Number of patients with *de novo* or biallelic *RNU2-2* variants in the PFMG and other cohorts. Details of other cohorts appear in Supplementary Table 7. Note that the variant numbers include multiple occurrences of the same variants. The number of distinct (‘unique’) variants in *RNU2-2* appear in brackets. Variants *in cis* with *de novo* or biallelic variants are not counted.

**Extended Data Fig. 2:**
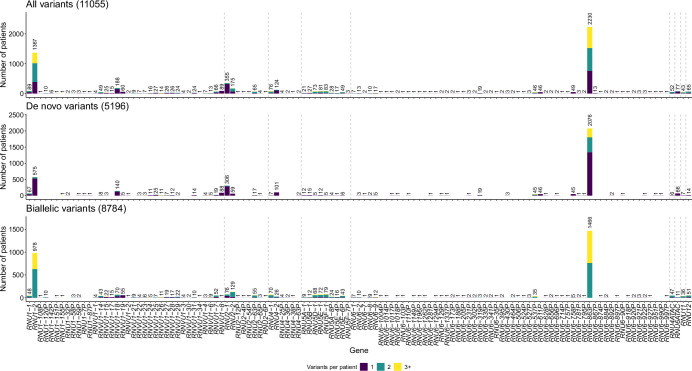
Distribution of variants across 200 putatively functional snRNA genes in the PFMG cohort. Supplementary Figure 1 shows the same distribution in the SeqOIA and Auragen subcohorts separately. Variants were found in 121 of the 200 genes.

**Extended Data Fig. 3:**
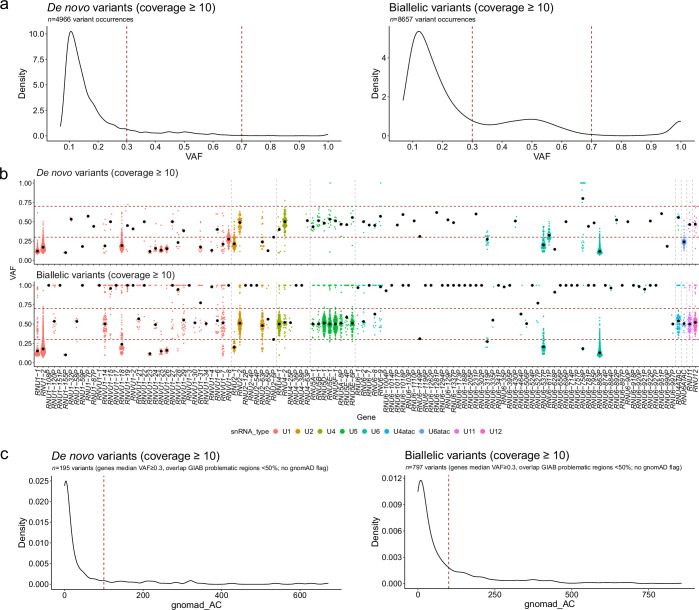
Variant allele fraction (VAF) distributions of snRNA variants in the aggregated PFMG cohort. **a**, VAF distribution of *de novo* and biallelic variants in snRNA genes with coverage ≥ 10. **b**, Detailed VAF distribution for snRNA genes harboring variants in the PFMG cohort. Supplementary Fig. 2 shows the same data in the SeqOIA and Auragen subcohorts separately. **c**, gnomAD allele count (AC) distribution of *de novo* and biallelic variants in snRNA genes (with coverage ≥ 10) in the PFMG cohort. Supplementary Fig. 3 shows the same data in the SeqOIA and Auragen subcohorts separately.

We next analyzed variants in the 200 potentially functional snRNA genes in the Plan France Médecine Génomique 2025 (PFMG) cohort^28^, which comprised short-read genome data from 34,329 patients with rare disorders (22,775 with NDDs). We focused on de novo and biallelic variants, hypothesizing roles in dominant or recessive monogenic disorders. After accounting for artefacts from short-read mapping and excluding low-quality variants (Supplementary Notes), 843 high-confidence variants (330 de novo, 551 biallelic) in 66 genes were identified in 616 patients (Extended Data Figs. 1–3 and Supplementary Figs. 1–4).

**Fig. 2:**
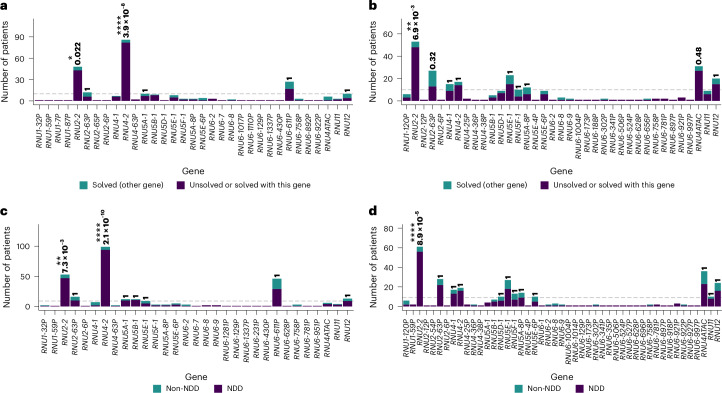
Identification of potential new snRNA gene–disease associations in the PFMG cohort. **a**,**b**, The cohort was divided into solved (*n* = 9,180) and unsolved (*n* = 20,735) cases for discovery analyses, with cases solved by variants in snRNAs with known disease association merged into the unsolved group. We compared the proportion of cases with rare variants (gnomAD allele count <100) between solved and unsolved groups for rare de novo variants (**a**) and rare biallelic variants (**b**). **c**,**d**, The cohort was divided into NDD (*n* = 22,775) and non-NDD (*n* = 11,554) cases for discovery analyses. We compared the proportion of cases with rare variants (gnomAD allele count <100) between NDD and non-NDD groups for rare de novo (**c**) and rare biallelic (**d**) variants. Two-sided Fisher’s tests were used to test statistical enrichment in unsolved versus solved cases for genes with at least ten patients carrying variants (**a**,**b**) and in NDD versus non-NDD cases for genes with at least nine such patients (**c**,**d**) (dashed gray line: minimum number of patients needed to reach statistical significance in the cohort). The number of patients per group and gene are shown in Supplementary Tables 2 and 4. Correction for multiple comparisons using Bonferroni was performed independently for unsolved versus solved cases and NDD versus non-NDD cases. Adjusted *P* values are shown above the bars. *P = 0.01–0.05; **P = 0.001–0.01; ****P < 0.0001.

**Extended Data Fig. 4:**
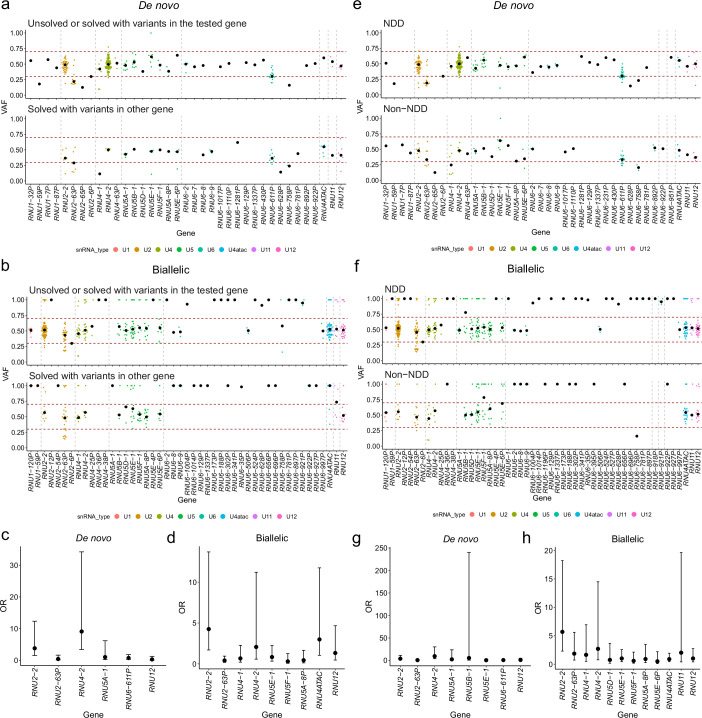
Identification of potential novel snRNA gene-disease associations in the PFMG cohort. **a-d**, The cohort was divided into solved (*n* = 9,180) and unsolved (*n* = 20,735) cases for discovery analyses, with cases solved by variants in snRNAs with known disease association merged into the unsolved group. We compared the proportion of cases with rare variants (gnomAD allele count < 100) between solved and unsolved groups. **a**,**b**, Variant allele fraction (VAF) distribution for rare *de novo* variants (**a**) and rare biallelic variants (**b**). **c**,**d**, Odds ratio (OR; dot) and OR 95% confidence interval (error bars) of unsolved versus solved cases for genes in which at least 10 patients had *de novo* (**c**) or biallelic (**d**) variants (minimum number needed to reach statistical significance in the cohort). **e**-**h**, The cohort was divided into NDD (*n* = 22,775) and non-NDD (*n* = 11,554) cases for discovery analyses. We compared the proportion of cases with rare variants (gnomAD allele count < 100) between NDD and non-NDD groups. **e**,**f**, Variant allele fraction (VAF) distribution for rare *de novo* variants (**e**) and rare biallelic variants (**f**). **g**,**h**, Odds ratio (OR; dot) and OR 95% confidence interval (error bars) of NDD versus non-NDD cases for genes in which at least 9 patients had *de novo* (**g**) or biallelic (**h**) variants (minimum number needed to reach statistical significance in the cohort). Two-sided Fisher’s test *P*-values are shown in Fig. 2. Supplementary Figs. 5, 6 respectively show the same analyses performed separately in the SeqOIA and Auragen subcohorts.

To identify possible new disease gene associations, we divided the cohort into solved (*n* = 9,180) and unsolved (*n* = 20,735) cases. Pathogenic (P) and likely pathogenic (LP) variants in disease-associated snRNA genes (*RNU4ATAC*, *RNU4-2*, *RNU5B-1*, *RNU2-2*) were curated manually and reassigned to the unsolved category, as they would have been before gene–disease association. We assessed both de novo and biallelic variants in the 66 genes focusing on rare variants (allele counts <100 in gnomAD v.3; Extended Data Fig. 3c). De novo variants were enriched significantly in *RNU4-2* and *RNU2-2* when comparing solved and unsolved cases (or gene-solved cases). For biallelic variants, *RNU2-2* showed significant enrichment across combined cohorts (*P* = 6.9 × 10^−3^, two-sided Fisher’s test), whereas *RNU4ATAC* reached significance only in the SeqOIA subcohort (Fig. 2a,b, Extended Data Fig. 4, Supplementary Fig. 5 and Supplementary Tables 2 and 3). We then compared NDD (*n* = 22,775) and non-NDD (*n* = 11,554) cases. In the entire PFMG cohort, de novo variants were enriched significantly in *RNU4-2* and *RNU2-2*, whereas biallelic variants were enriched only in *RNU2-2* (*P* = 8.9 × 10^−5^, two-sided Fisher’s test) (Fig. 2c,d, Extended Data Fig. 4, Supplementary Fig. 6 and Supplementary Tables 4 and 5). These results indicate that *RNU2-2* variants probably contribute to both dominant and recessive NDDs, similar to *RNU4-2*^21,22^.

### *RNU2-2* variants as a cause of dominant and recessive disorders

**Fig. 3:**
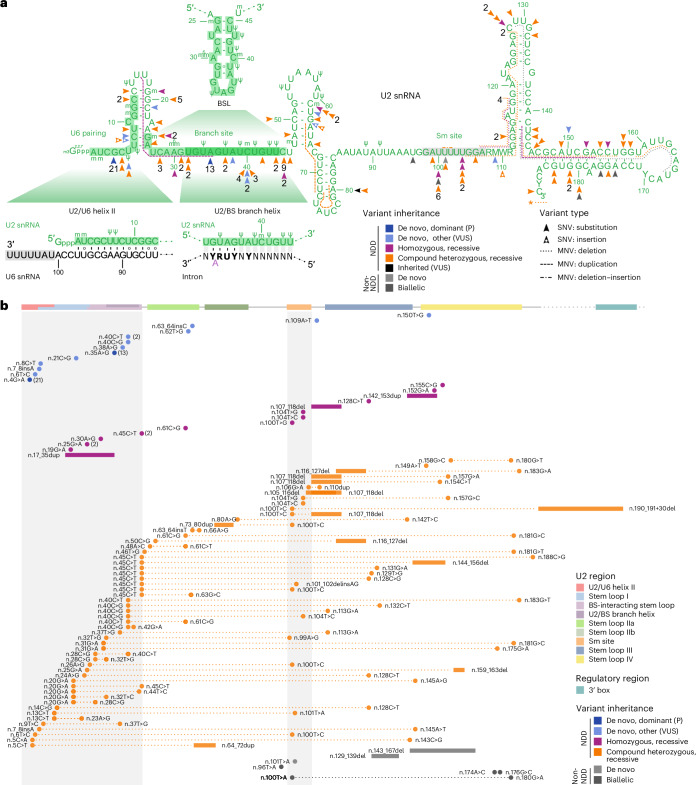
Overview of *RNU2-2* variants identified in this study. **a**, Two-dimensional predicted structure of U2-2 snRNA showing structural and functional domains. Arrowheads indicate point variants identified in this study. Variants are colored according to their inheritance. Dark blue: de novo, dominant (n.4G>A or n.35A>G); light blue: de novo other (VUS); orange: compound heterozygous, recessive; purple: homozygous, recessive. The numbers in black represent the count of patients with each variant, for variants identified in more than one family. Other variant types are shown with dotted (deletions), dashed (duplications) or dotted-dashed (indels) lines. Asterisk, deletion encompassing the 3′ box. The nucleotide differences between *RNU2-2* and *RNU2-1* are shown using IUPAC codes. Green numbers refer to the numbering of U2-2 nucleotides (nt). Ψ, pseudouridine; m, 2′-O-methyl residues; m6, N6-methyladenosine; ^2,2,7^m3Gppp, 2,2,7-trimethylguanosine cap. Green-shaded regions: functional domains of U2 involved in spliceosomal activity: U2/U6 helix II (nt 1–13); BSL (nt 25–45) and U2/BS branch helix (nt 32–44). Gray-shaded region: Sm site. **b**, Locations of variants on the *RNU2-2* gene, with different domains of the snRNA highlighted on the schematic, extending to the 3′ box (regulatory region). Note the clustering of variants in the U2/U6 helix, BSL regions and Sm site (gray-shaded), as well as preferential associations of compound heterozygous variants. Variants are ordered by inheritance mode and by the position of the first variant on the snRNA. Variant coloring as in **a**.

To refine the search for pathogenic variants, we reanalyzed the PFMG cohort restricting only the homozygote frequency (<3 in gnomAD v.3, <5 in internal databases), then applied filters on allele frequencies in All of Us (AoU) database (AC < 50 for de novo and <200 for biallelic variants) (Supplementary Fig. 7). Using these criteria, 42 unrelated patients had rare de novo *RNU2-2* variants (Supplementary Table 6). Twenty-one unrelated patients and one monozygotic twin harbored previously reported pathogenic alleles: n.4G>A (*n* = 11) and n.35A>G (*n* = 11 including the twin). One patient with n.35A>G was mosaic, and deep-targeted sequencing (>2,000×) confirmed mutant allele fractions of 12% (678 of 5,218 reads) in blood, 20% (1,181 of 5,659 reads) in urine and 25% (574 of 2,267 reads) in buccal cells. In addition, n.4G>A was observed in a singleton. The remaining 21 patients had other de novo variants (Fig. 3 and Supplementary Notes). In 7 of 21 individuals (with n.5C>A, n.6T>C, n.7_8insA, n.31G>A, n.37T>G (*n* = 2) and n.40C>T), further examination revealed a second rare variant that was always in *trans*, suggesting recessive inheritance. In contrast, a single patient with n.4G>A had a second variant in *trans* (n.80A>G; Supplementary Table 6). In addition to these seven cases, 45 probands had rare biallelic variants in the PFMG cohort.

To expand the *RNU2-2* variant spectrum, we reanalyzed available genome data and/or performed targeted sequencing of *RNU2-2* in 5,456 people with NDDs and identified additional cases with *RNU2-2* variants through *seqr*^29^ (Supplementary Table 7). This large international collaborative effort revealed 34 additional unrelated patients: 13 had monoallelic variants, including 9 patients with n.4G>A (8 de novo and 1 nonmaternal), 3 n.35A>G variants (2 de novo) and 1 with de novo n.38A>G; 21 families had biallelic variants (Extended Data Fig. 1b).

**Fig. 4:**
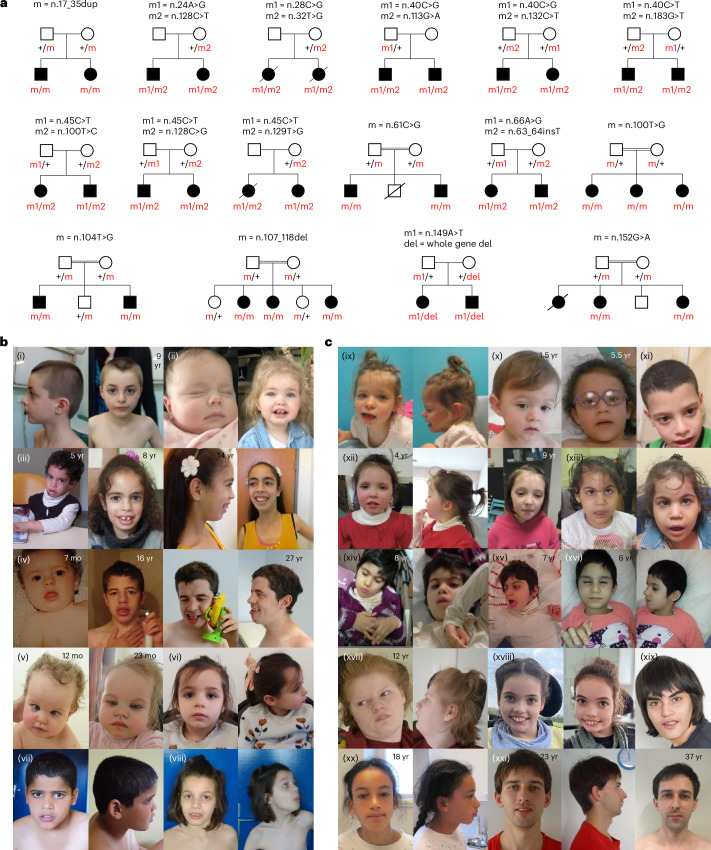
Variant segregation and facial features associated with *RNU2-2* variants. **a**, Segregation of biallelic variants in the 16 families with at least two affected siblings. **b**, Facial photographs of people with monoallelic *RNU2-2* variants: (i)–(iv) n.4G>A; (v)–(vii), n.35A>G; (viii) n.35A>G in mosaic. **c**, Facial photographs of individuals with biallelic *RNU2-2* variants. (ix) maternal n.104T>C/paternal gene deletion; (x) maternal n.40C>G/paternal n.42G>A; (xi) homozygous n.61C>G; (xii) paternal n.48A>C/maternal n.61C>T; (xiii) homozygous n.152G>A; (xiv)–(xvi) homozygous n.107_118del in three affected sisters; (xvii) de novo n.6T>C/maternal n.100T>C; (xviii) maternal n.20G>A/paternal n.28C>G; (xix) homozygous n.100T>G; (xx) homozygous n.128C>T; (xxi) nonmaternal n.13C>T/maternal n.101T>A. Consent was obtained for publication of facial photographs. mo, months; yr, years.

**Extended Data Fig. 5:**
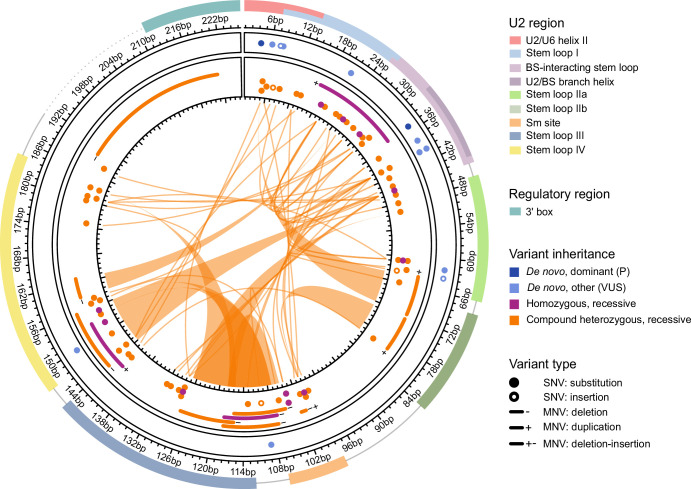
Circos plot depicting preferential associations of biallelic variants. This scheme suggests that, in affected individuals with compound heterozygote variants, variants preferentially co-occur with one affecting the 5′ domain and the other the 3′ domain, suggesting domain-specific combinatorial effects. Variant coloring is the same as in Fig. 3a.

**Extended Data Fig. 6:**
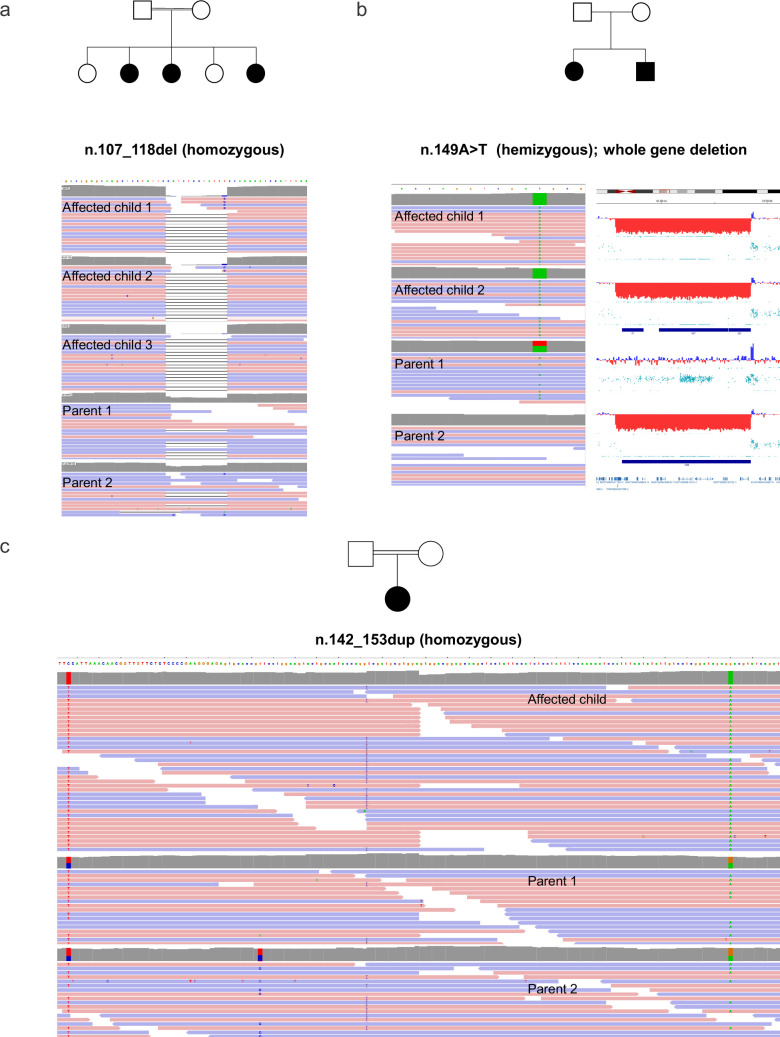
Examples of biallelic variants identified by genome sequencing in the PFMG cohort, with variant segregation consistent with recessive inheritance. **a**, Integrative Genomics Viewer (IGV) screenshots of BAM file alignments showing segregation of the *RNU2-2* n.127_118del variant in the homozygous state in three affected sisters, with both parents carrying the variant in heterozygous state. **b**, Left: IGV screenshot displaying the hemizygous *RNU2-2* n.149A>T variant in two affected siblings and their heterozygous mother. Right: Copy-number analysis of genome sequencing data revealing a 642-kb deletion (hg38: chr11:62,781,800-63,424,493) encompassing *RNU2-2* and 31 additional genes, present in the two affected siblings and their father. **c**, IGV screenshot showing the presence of the *RNU2-2* n.142_153dup variant in homozygous state in the affected child and in heterozygous state in both parents.

Combined with the PFMG cohort, we report a total of 141 patients from 122 unrelated families carrying 96 distinct point variants in *RNU2-2* that we classified using American College of Medical Genetics and Genomics criteria (Supplementary Table 8) and two partial-/whole-gene deletions: 35 patients (including the twin) had the dominant n.4G>A and n.35A>G pathogenic variants; 15 patients had another single de novo heterozygous variant; 91 patients from 73 unrelated families exhibited biallelic variants. Of these, 54 carried compound heterozygous variants, 17 had homozygous variants and 2 harbored hemizygous variants associated in trans with a complete or partial gene deletion (Fig. 3 and Extended Data Fig. 5). In 16 families, one or more siblings were also affected, with both variants cosegregating with the disease in affected siblings (Fig. 4a and Extended Data Fig. 6). Twenty-two recessive variants were found in at least two unrelated families, including n.20G>A, n.40C>G, n.40C>T, n.45C>T, n.100T>C, n.107_118del in at least five families and 16 additional variants in two or three families (Supplementary Notes and Supplementary Table 8).

Genome data were available for 110 of the 122 index cases. A single person had a de novo nonsense variant in *GATA3*, partially explaining his phenotype; all others had remained unsolved. Overall, 104 of the 110 patients presented with a NDD phenotype. Of the six remaining cases, three harbored de novo monoallelic variants (one person with cancer, one fetus with a cerebral malformation and one non-NDD patient) and three had biallelic variants (one terminated fetus and two non-NDD patients, including the person with the *GATA3* variant). In the PFMG cohort, NDD was the predominant presentation, observed in 82 of 88 patients (*P* = 1.89 × 10^−18^, one-tailed binomial test; 95% confidence interval, 0.87–1.00).

Twenty-six de novo variants could be phased reliably: 16, including all phaseable occurrences of the pathogenic n.4G>A (*n* = 8) and n.35A>G (*n* = 2), along with n.5C>A, n.6T>C and n.7_8insA (*n* = 2), n.31G>A and n.37T>G were phased to the maternal allele. The remaining ten (n.6T>C; n.21C>G; n.32T>G; n.37T>G; n.40C>T, *n* = 3; n.62T>G; n.129_139del; n.143_167del) arose de novo on the paternal allele (Supplementary Table 6).

### Dominant and recessive *RNU2-2* NDDs share overlapping features

We next aimed to delineate the clinical spectrum associated with both dominant and recessive *RNU2-2* disorders. Detailed clinical data were collected for 112 patients (55 female, 57 male), including 20 with n.4G>A, 12 with n.35A>G, and 7 with another monoallelic de novo variant, as well as 73 patients from 55 unrelated families with biallelic inheritance (Table 1 and Supplementary Tables 9–11). The median age at inclusion in the study was 13 years (range: 0 (fetus) to 46 years).

**Table 1:** Overview of clinical features in patients with monoallelic and biallelic *RNU2-2* variants

Group	n.4G>A	n.35A>G	Monoallelic	Biallelic	Total
Number of patients	20	12	32	73	105
Epilepsy	19 of 20 (95%)	9 of 12 (75%)	28 of 32 (88%)	60 of 72 (83%)	88 of 104 (85%)
Generalized seizures	9 of 16 (56%)	6 of 8 (75%)	15 of 24 (62%)	39 of 44 (89%)	54 of 68 (79%)
Tonic-clonic seizures	6 of 15 (40%)	4 of 8 (50%)	10 of 23 (43%)	28 of 37 (76%)	38 of 60 (63%)
Myoclonic seizures	4 of 15 (27%)	3 of 9 (33%)	**7 of** **24 (29%)**	**30 of** **40 (75%)**	37 of 64 (58%)
Tonic seizures	13 of 16 (81%)	6 of 8 (75%)	19 of 24 (79%)	13 of 32 (41%)	32 of 56 (57%)
Focal seizures	7 of 15 (47%)	3 of 9 (33%)	10 of 24 (42%)	19 of 31 (61%)	29 of 55 (53%)
Nonmotor seizures (absences)	9 of 16 (56%)	1 of 8 (12%)	10 of 24 (42%)	22 of 39 (56%)	32 of 63 (51%)
Spasms	5 of 16 (31%)	1 of 8 (12%)	6 of 24 (25%)	15 of 35 (43%)	21 of 59 (36%)
Atonic seizures	3 of 15 (20%)	3 of 8 (38%)	6 of 23 (26%)	13 of 33 (39%)	19 of 56 (34%)
Clonic seizures	7 of 16 (44%)	1 of 8 (12%)	8 of 24 (33%)	9 of 27 (33%)	17 of 51 (33%)
Generalized and focal seizures	7 of 16 (44%)	4 of 8 (50%)	11 of 24 (46%)	7 of 46 (15%)	18 of 70 (26%)
Hemicorporal seizures	6 of 15 (40%)	1 of 8 (12%)	7 of 23 (30%)	4 of 27 (15%)	11 of 50 (22%)
Febrile seizures	**11 of** **16 (69%)**	**0 of** **10 (0%)**	11 of 26 (42%)	6 of 52 (12%)	17 of 78 (22%)
Status epilepticus	15 of 18 (83%)	5 of 7 (71%)	20 of 25 (80%)	40 of 57 (70%)	60 of 82 (73%)
Pharmoresistance	8 of 17 (47%)	6 of 8 (75%)	14 of 25 (56%)	18 of 51 (35%)	32 of 76 (42%)
Age of seizure onset: <3 years	15 of 19 (79%)	7 of 9 (78%)	22 of 28 (79%)	50 of 60 (83%)	72 of 88 (82%)
Age of seizure onset: >3 years	4 of 19 (21%)	2 of 9 (22%)	6 of 28 (21%)	10 of 60 (17%)	16 of 88 (18%)
Daily seizures	11 of 15 (73%)	1 of 8 (12%)	12 of 23 (52%)	29 of 51 (57%)	41 of 74 (55%)
Severe intellectual disability	15 of 19 (79%)	10/10 (100%)	25 of 29 (86%)	50 of 68 (74%)	75 of 97 (77%)
Severe developmental delay	13 of 20 (65%)	12 of 12 (100%)	25 of 32 (78%)	51 of 70 (73%)	76 of 102 (75%)
No language	15 of 20 (75%)	8 of 11 (73%)	23 of 31 (74%)	41 of 62 (66%)	64 of 93 (69%)
Autism spectrum disorder	14 of 20 (70%)	9 of 11 (82%)	23 of 31 (74%)	31 of 60 (52%)	54 of 91 (59%)
Hospitalization	11 of 18 (61%)	5 of 12 (42%)	16 of 30 (53%)	37 of 61 (61%)	53 of 91 (58%)
Stereotypies	14 of 19 (74%)	6 of 11 (55%)	20 of 30 (67%)	32 of 61 (52%)	52 of 91 (57%)
Other behavioral anomalies	6 of 17 (35%)	8 of 11 (73%)	14 of 28 (50%)	31 of 58 (53%)	45 of 86 (52%)
Feeding issues, gastro-intestinal reflux	8 of 18 (44%)	4 of 12 (33%)	12 of 30 (40%)	38 of 67 (57%)	50 of 97 (52%)
Sleep disorders	11 of 19 (58%)	1 of 11 (9%)	12 of 30 (40%)	37 of 64 (58%)	49 of 94 (52%)
MRI abnormalities	6 of 18 (33%)	3 of 10 (30%)	9 of 28 (32%)	38 of 63 (60%)	47 of 91 (52%)
Constipation	9 of 18 (50%)	3 of 11 (27%)	12 of 29 (41%)	31 of 64 (48%)	43 of 93 (46%)
Neonatal hypotonia	9 of 19 (47%)	5 of 12 (42%)	14 of 31 (45%)	29 of 71 (41%)	43 of 102 (42%)
Dysmorphic features	9 of 19 (47%)	6 of 12 (50%)	15 of 31 (48%)	24 of 67 (36%)	39 of 98 (40%)
Regression	5 of 19 (26%)	4 of 11 (36%)	9 of 30 (30%)	28 of 66 (42%)	37 of 96 (39%)
Movement disorder	3 of 15 (20%)	0 of 8 (0%)	3 of 23 (13%)	26 of 53 (49%)	29 of 76 (38%)
Hypersialorrhea/drooling	9 of 18 (50%)	4 of 12 (33%)	13 of 30 (43%)	21 of 64 (33%)	34 of 94 (36%)
Short stature	8 of 20 (40%)	5 of 11 (45%)	13 of 31 (42%)	19 of 63 (30%)	32 of 94 (34%)
Eyes/vision abnormalities	3 of 18 (17%)	3 of 12 (25%)	6 of 30 (20%)	25 of 64 (39%)	31 of 94 (33%)
Joint hyperlaxity	5 of 18 (28%)	3 of 12 (25%)	8 of 30 (27%)	22 of 62 (35%)	30 of 92 (33%)
Microcephaly	4/18 (22%)	3 of 11 (27%)	7 of 29 (24%)	22 of 64 (34%)	29 of 93 (31%)
Ataxia	7 of 18 (39%)	5 of 9 (56%)	12 of 27 (44%)	11 of 51 (22%)	23 of 78 (29%)
Failure to thrive/growth retardation	5 of 19 (26%)	3 of 12 (25%)	8 of 31 (26%)	19 of 62 (31%)	27 of 93 (29%)
Bone/skeletal anomalies	4 of 18 (22%)	2 of 12 (17%)	6 of 30 (20%)	18 of 65 (28%)	24 of 95 (25%)
Hyperventilation	3 of 18 (17%)	0 of 11 (0%)	3 of 29 (10%)	13 of 64 (20%)	16 of 93 (17%)
Prenatal findings	1 of 18 (6%)	2 of 12 (17%)	3 of 30 (10%)	9 of 65 (14%)	12 of 95 (13%)

MRI, magnetic resonance imaging. Clinical features are listed by frequency across the entire patient cohort, with significant differences between groups shown in bold. Detailed clinical features and statistical comparisons for all three groups, including patients with de novo variants lacking a second pathogenic allele, are shown in Supplementary Table 10 (index cases only) and Supplementary Table 11 (index cases + siblings).

Overall, considering only patients with dominant pathogenic (*n* = 32, including the twin) and biallelic variants (*n* = 73), all patients with available data had developmental delay, and all older than 3 years had intellectual disability (ID) except one with developmental delay without ID, showing fragile visuoconstructive reasoning (n.20G>A/n.145A>G). Severe/profound ID was most frequent (75 of 97, 77%), followed by moderate (17 of 97, 18%) and mild (4 of 97, 4%) ID. Epilepsy occurred in 85% patients (88 of 104), with identical rates in dominant (28 of 32, 88%) and recessive (60 of 72, 83%) cases. Age at seizure onset ranged from 8 weeks to 16 years (median: 1.5 years); 82% (72 of 88) had seizure onset between 8 weeks and 3 years (monoallelic: 22 of 28 biallelic: 50 of 60), while 18% (16 of 88) had seizures after 3 years (monoallelic: 6 of 28; biallelic: 10 of 60). Seizure types were variable. In biallelic families, 39 of 44 (89%) had generalized seizures, whereas in monoallelic cases, 19 of 24 (79%) had focal seizures, including hemicorporal seizures in 7 of 23 (30%). Generalized tonic-clonic seizures occurred in 38 of 60 patients (63%; biallelic 28 of 37, monoallelic 10 of 23), myoclonic seizures in 75% versus 29%, epileptic spasms in 43% versus 25%, and absence seizures in 32 of 63 patients (51%; monoallelic 10 of 24, biallelic 22 of 39). Patients were treated according to their seizure type, as is standard clinical practice, with 60 of 82 patients (73%; monoallelic 20 of 25, biallelic 40 of 57) exhibiting drug resistance. Patients with epilepsy met the criteria for developmental and epileptic encephalopathy. Among the 16 patients without epilepsy, 4 had dominant pathogenic variants (3 with n.35A>G, ages 29 months–4 years; 1 with n.4G>A, age 6 years). Twelve (ages 2.6–39 years) had biallelic variants, including three with n.40C>T. One terminated fetus (n.174A>C/n.176G>C) had a polymalformation syndrome.

**Extended Data Fig. 7:**
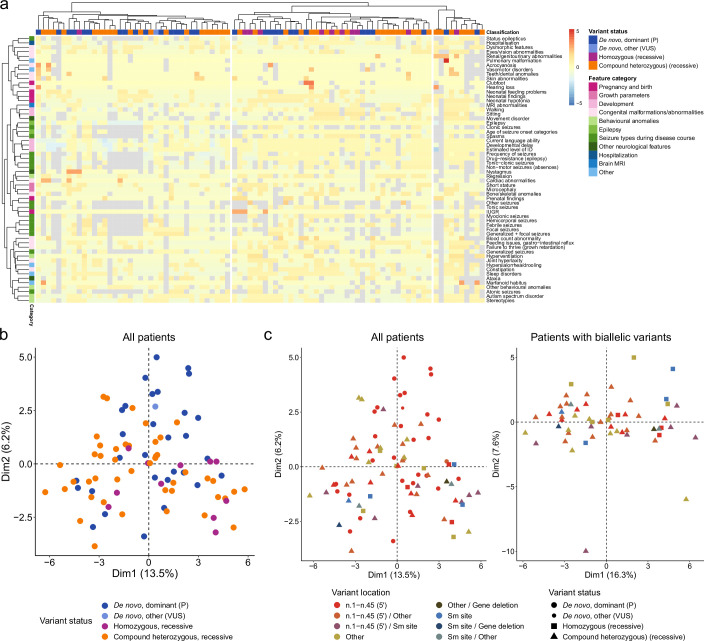
Patients with dominant and recessive *RNU2-2* NDDs have similar clinical features without obvious genotype-phenotype correlations. **a**, Hierarchical clustering of the clinical features (*n* = 60, rows) of patients with *de novo* dominant or biallelic *RNU2-2* variants (*n* = 87, columns). Categorical data was converted to 0-1 scale, and values were *Z*-score scaled for each row. Blue-yellow-red scale depicts *Z*-scores. Lower values indicate a more favorable phenotype, while higher values represent a more severe phenotype. Missing values are shown in grey. Columns are colored based on the variant classification. Dark blue: *de novo*, dominant (n.4G>A or n.35A>G; *n* = 30); light blue: *de novo* other (VUS; *n* = 1); orange: compound heterozygous, recessive (*n* = 45); purple: homozygous, recessive (*n* = 11). **b**, Principal component analysis of clinical features in *RNU2-2* variant carriers taking the variant status into account. Variant coloring is the same as in **a**. **c**, Principal component analysis of clinical features in *RNU2-2* variant carriers, considering variant type, location, and combinations. PCA on the left include all patients while PCA on the right include only patients with biallelic variants. Missing values were imputed as 0.

Clinical presentations varied within each group (n.4G>A, n.35A>G and biallelic variants) but, overall, the clinical spectrums were similar, with no clear genotype–phenotype correlations (Table 1 and Extended Data Fig. 7). Myoclonic seizures were more prevalent in biallelic than monoallelic cases (30 of 40, 75% versus 7 of 24, 29%; two-sided Fisher’s test *P* = 0.028). Febrile seizures occurred in biallelic (6 of 52, 12%) and monoallelic (11 of 26, 42%) cases but were preponderant in n.4G>A cases (11 of 16, 69%), and absent in n.35A>G carriers (0 of 10, *P* = 0.027; two-sided Fisher’s test). Movement disorders seemed more frequent in biallelic (26 of 53, 49%) than in dominant (3 of 23, 13%) cases (not significant after correction). The most severe phenotypes, including all eight reported deaths (ages 3–19 years; median 15 years) due to respiratory, infectious or epilepsy-related complications, were restricted to biallelic patients. Despite intragroup variability, ID severity, seizures and movement disorders were consistent within families, suggesting that variant combinations contribute to phenotypic variability of recessive NDDs. Minor dysmorphic features, such as broad forehead, midface hypoplasia, a large open mouth, a small chin and down-slanting palpebral fissures were common in patients with available photographs (Fig. 4b,c).

Heterozygous parents of patients with biallelic variants were generally unaffected, except in five cases: a mother with n.5C>T and a father with n.32T>C had childhood epilepsy; a mother with n.61C>T had epilepsy; both parents carrying n.17_35dup were affected, the father with ID and the mother with ID and epilepsy.

Seven patients with monoallelic de novo variants other than n.4G>A and n.35A>G had highly variable phenotypes: four patients had mild ID without epilepsy (*n* = 3) or moderate ID with epilepsy (*n* = 1); one died at 6 days from status epilepticus; one fetus was terminated due to semi-lobar holoprosencephaly. The remaining patient had hearing loss and chronic bronchopulmonary disease.

### Predicted impact of *RNU2-2* variants

To assess the impact of *RNU2-2* variants, we examined their distribution across U2 structural and functional domains and mapped them onto published U2 snRNP and spliceosome structures^30,31^. U2-2 can be divided into three functional domains, each remodeled to different extents throughout the splicing cycle and snRNP biogenesis. The 5′ domain forms four partially mutually exclusive structures: (1) the intramolecular branch-point-interacting stem–loop (BSL; n.25–45)^32^, (2) stem–loop I (SLI; n.7–26)^33^, (3) the intermolecular U2/U6 helix II (n.1–13)^1^ and (4) the U2/U6 helix Ia and Ib (n.20–28)^34^. The branch interacting region oscillates between BSL conformation and stable intron binding through formation of the U2/BS branch helix (n.32–44)^1,35^. The 3′ end domain encompasses five structural elements: stem loops IIa (SLIIa; n.47–66)^36^ and IIb (SLIIb; n.68–84)^32^, the Sm binding site (n.98–107)^37^ and 3′ stem loops III and IV (SLIII; n.112–144/SLIV; n.147–184)^38,39^.

**Fig. 5:**
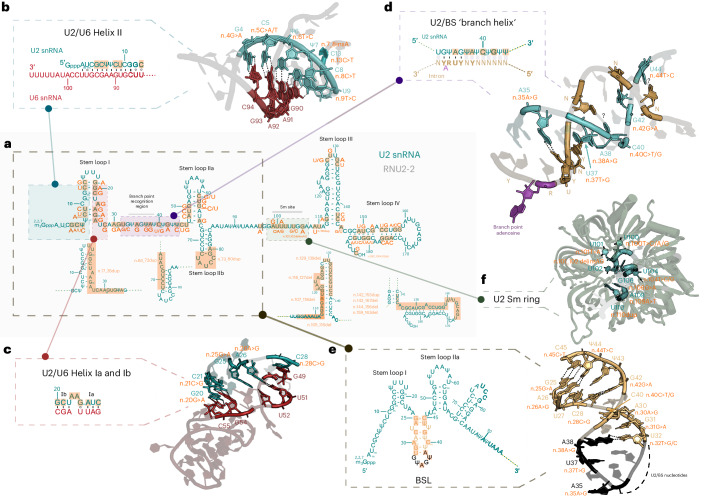
Possible functional impact of *RNU2-2* variants. Overview of *RNU2-2* variants identified in this study. **a**, Two-dimensional structure of the U2 snRNA (green). Orange boxes indicate variants from this study, with point mutations and single nucleotide insertions represented on the graph along with their respective changes. ^2,2,7^m3Gppp, 2,2,7-trimethylguanosine cap; Ψ, pseudouridine; m, 2′-O-methyl residues. **b**, Zoom-in box representing the two-dimensional predicted structure of the U2 (teal) and U6 (red) snRNAs U2/U6 helix II during formation of precatalytic spliceosome. Interactions stabilizing this structure as well as mutations potentially affecting its stability are represented on coordindates (PDB 5XJC). **c**, Zoom-in box representing the two-dimensional predicted structure of U2/U6 helices Ia and Ib. Nucleotides involved in the structure and associated with pathological variants are represented (PDB 5XJC). **d**, Close-up of U2/BS branch helix where the two-dimensional predicted structure of the interaction between branch-point recognition region of U2 snRNA and the intronic BS (light brown) is represented. The branch-point adenosine is highlighted in purple. ‘YNYURAY,’ consensus BS in metazoans: Y, pyrimidine; N, any nucleotide; R, purine. The mutations in this region are depicted on the structure (PDB 5XJC). **e**, Zoom-in box representing the two-dimensional predicted structure of U2 snRNA within the 17S snRNP with the BSL highlighted. In black are nucleotides involved directly on the BS recognition. The structure of the BSLwithin the 17S U2 snRNP is represented (PDB 7Q3L). **f**, Close-up of the U2 snRNA Sm ring (teal), with nucleotides with mutations close to, or within, it marked in orange (PDB 5XJC).

*RNU2-2* variants at the 5′ end probably destabilize U2/U6 helix II by disrupting key Watson–Crick base pairs^33^: n.4G>A breaks the G4–C94 pair, n.5C>A/T disrupts C5–G93, n.6T>C alters U6–A92 and n.8C>T the C8–G90 (Fig. 5). These changes may impair tri-snRNP recruitment to the prespliceosome and reduce splicing efficiency^40–42^. n.7_8insA, n.8C>T, n.9T>C, n.13C>T, n.14C>G, n.19G>A, n.20G>A, n.21C>G, n.23A>G, n.24A>G, n.25G>A and n.26A>G would affect SL1 stability and directly or indirectly alter U2/U6 helix II formation. Furthermore, n.20G>A, n.21C>G, n.26A>G and n.28C>G may destabilize helices Ia and Ib by replacing Watson–Crick base pairs with noncanonical pairs, perturbing active site formation.

Some variants within the BSL probably affect its stability. For instance, n.40C>T or n.30A>G may create a Watson–Crick base pair between positions 30 and 40 that hyperstabilizes the BSL, which was linked previously to reduced splicing fidelity in yeast^32^. Likewise, n.40C>G may stabilize the BSL by creating a G–A base pair replacing the C–A mismatch^43^. Conversely, n.25G>A, n.26A>G, n.28C>G, n.32T>C/G, n.38A>G, n.42G>A, n.44T>C and n.45C>T may disrupt BSL integrity^44^. Notably, n.35A>G alters the invariant A35 of U2 that pairs with the conserved U upstream of the branch site (BS), which may increase pairing flexibility, promoting cryptic BS usage^45^ (Fig. 5). Besides, n.28C>G, which may affect both BSL formation and the active site, is a recurrent somatic variant detected in cancers^24^.

In contrast, variants in SLIIa, the Sm site and the 3′ stem loops SLIII/SLIV probably impair U2 snRNP biogenesis and nuclear import, destabilizing U2 and preventing proper spliceosome assembly. Likewise, n.190_191+30del would perturb proper 3′ end processing. These changes may behave effectively as null alleles such as gene deletions, as the affected U2 molecules may fail to reach functional spliceosomes.

### Impact of *RNU2-2* variants on lymphocyte transcriptome

**Fig. 6:**
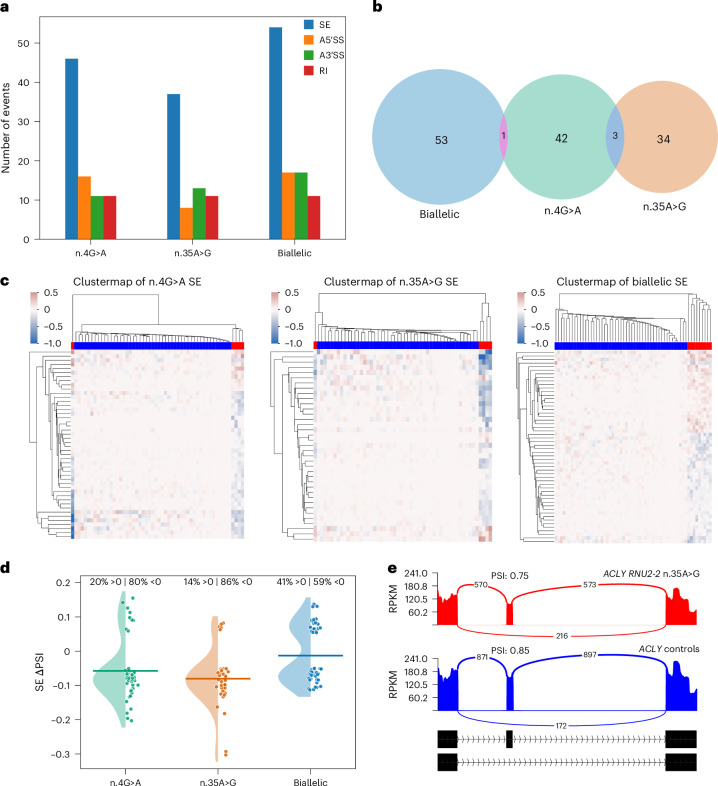
Variant-specific alternative splicing perturbations in *RNU2-2* variant carriers. **a**, Number of significant alternative splicing events (ΔPSI > 0.05) detected using rMATS-turbo and linear regression (*P* < 0.01), comparing *RNU2-2* patients (dominant: n.4G>A, *n* = 5; n.35A>G, *n* = 5; biallelic: *n* = 9) to 49 controls. Splicing categories are color-coded: SE (blue), alternative 5′SS (A5′SS, orange), alternative 3′SS (A3′SS, green) and RI (red). **b**, Venn diagram showing minimal overlap of exon skipping events shared among dominant (n.4G>A, n.35A>G) and recessive (biallelic) variant groups. **c**, Clustermaps of PSI value residuals after linear regression for SE events of patients carrying n.4G>A (left, *n* = 5), n.35A>G (middle, *n* = 5) and biallelic (right, *n* = 9) variants. Blue: controls; red: *RNU2-2* cases. **d**, Distribution of ΔPSI across the three conditions. Raincloud plots show the density of ΔPSI values (half-violins), individual splicing events (points) and mean ΔPSI (horizontal bars). Percentages above each group indicate the proportion of events with increased (ΔPSI > 0) or decreased (ΔPSI < 0) exon inclusion. **e**, Sashimi plots illustrating an SE isoform shift in *ACLY* observed in *RNU2-2* n.35A>G variant carriers compared to controls. RPKM, reads per kilobase of transcript per million mapped reads.

We showed previously that pathogenic *RNU4-2* variants lead to splicing defects affecting primarily alternative 5′SS, detectable in cultured lymphocytes^18^. Here we investigated splicing alterations in 19 people with either dominant (n.4G>A, *n* = 5; n.35A>G, *n* = 5) or recessive (*n* = 9) *RNU2-2* variants and compared them with data of 49 patients with other disorders (controls). Using the same approach as for *RNU4-2*, we found no consistent splicing signature among *RNU2-2* variant carriers. To increase the specificity of our analysis, we applied a linear regression on percent-spliced-in (PSI) values (ΔPSI > 0.05), integrating age, sex and cell composition as covariates. Significant alternative splicing events (*P* < 0.01) were detected for 5′SS, alternative 3′ splice sites (3′SS), skipped exons (SEs) and retained introns (RIs). SEs represented the main effect in both dominant (n.4G>A and n.35A>G) and recessive *RNU2-2* (Fig. 6a) cases but showed minimal overlap between all three conditions (Fig. 6b). Clustering of PSI residuals for SE events distinguished *RNU2-2* variant carriers successfully from controls (Fig. 6c and Supplementary Tables 12–14). Analysis of ΔPSI distributions revealed a global decrease in exon inclusion (that is, increased exon skipping) in dominant cases, whereas recessive cases exhibited both increased and decreased exon skipping to a similar extent (Fig. 6d). All SE events were also present among controls (Fig. 6e), indicating that *RNU2-2* variants induce subtle quantitative perturbations in alternative splicing rather than generating new splice junctions in lymphocytes.

### Impact of *RNU2-2* variants on blood methylome

**Fig. 7:**
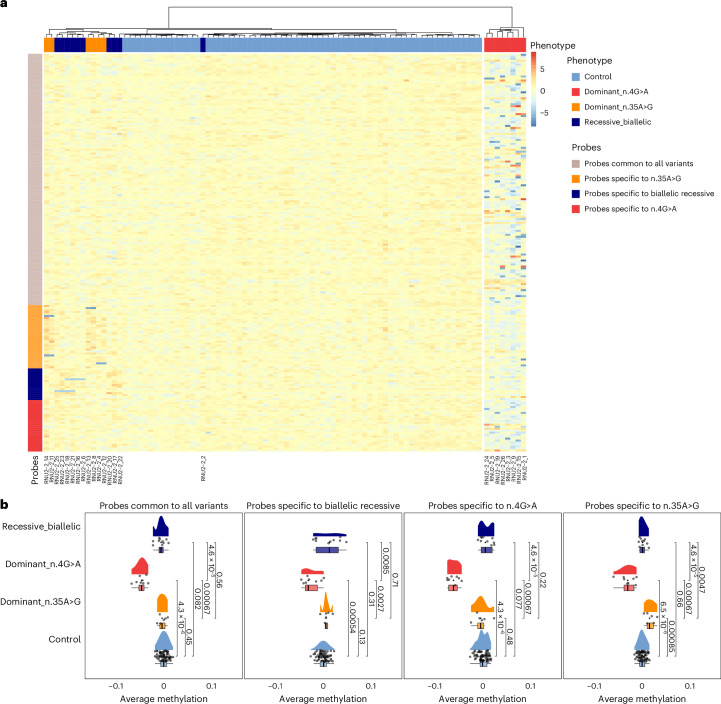
Methylation profiles across differentially methylated probes in *RNU2-2* variant carriers and normal controls. In all panels, controls (*n* = 68) are shown in light blue, dominant n.4G>A carriers (*n* = 8) in red, dominant n.35A>G carriers (*n* = 6) in orange and recessive biallelic cases (*n* = 10) in dark blue. **a**, Heatmap of adjusted methylation levels at differentially methylated CpGs. Selected probes are represented in rows whereas patients and normal controls are represented in columns after hierarchical clustering of their methylation profiles. Probes are grouped according to their association pattern: common to all variants, specific to n.35A>G, specific to biallelic recessive variants and specific to n.4G>A. Samples are annotated by type (control, dominant n.4G>A, dominant n.35A>G, recessive biallelic) but sample clustering was unsupervised and blind to this classification. **b**, Raincloud plots showing the average methylation level per sample within each probe association pattern. Normal controls and variant carriers display significantly different distributions, with *P* values from two-sided Wilcoxon rank-sum tests indicated on the plots. The number of individuals per group is the same as in **a**. Boxplot elements are as follows: centerline, median; box limits, upper and lower quartiles; whiskers, 1.5× interquartile range.

**Extended Data Fig. 8:**
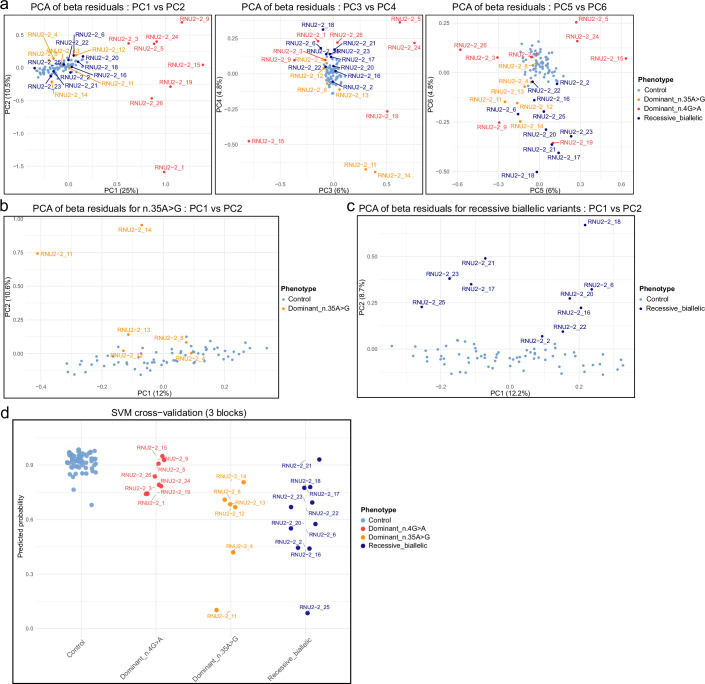
Principal component analysis and classification performance of the *RNU2-2* episignature. In all panels, controls are shown in light blue, dominant n.4G>A carriers in red, dominant n.35A>G carriers in orange, and recessive biallelic cases in dark blue. **a**, PCA of adjusted methylation levels at differentially methylated positions (*n* = 201) up to component 6, after correction for expected baseline methylation level based on age at sampling, sex and estimated blood cell counts (*n* = 92 individuals in total) showing separation of dominant n.4G>A carriers from normal controls on axis 1, and the weaker separation between dominant n.35A>G carriers or recessive biallelic cases from normal controls along axis 6. The percentage of variance explained is provided for each component within the axis title. **b**, PCA of adjusted methylation levels at differentially methylated positions (*n* = 201), after correction for expected baseline methylation level based on age at sampling, sex and estimated blood cell counts on the restriction to normal controls and carriers of the dominant n.35A>G variant along the first two principal components (*n* = 74 individuals in total). The percentage of variance explained is provided for each axis. **c**, PCA of adjusted methylation levels at differentially methylated positions (*n* = 201), after correction for expected baseline methylation level based on age at sampling, sex and estimated blood cell counts on the restriction to normal controls and carriers of recessive biallelic variants along the first two principal components (*n* = 78 individuals in total). The percentage of variance explained is provided for each axis. **d**, Predicted probabilities from a three-block cross-validation using a four-class SVM classifier (control, dominant n.4G>A, dominant n.35A>G, recessive biallelic).

We next analyzed DNA methylation profiles in 24 patients with *RNU2-2* variants (8 with n.4G>A, 6 with n.35A>G and 10 with biallelic variants) and compared them with 68 controls to identify potential episignatures, using a similar methodology to *RNU4-2*^18^. Cases and controls were matched for age at sampling, and analyses also corrected for age, sex and cell composition. Consistent with transcriptomic findings, patient groups seemed heterogeneous, limiting statistical power for variant-specific analyses. We then implemented a combined model simultaneously evaluating all three variant types while allowing for both shared and variant-specific effects. Overall, methylation signatures associated with *RNU2-2* variants were subtle, heterogeneous and weaker than reported previously for *RNU4-2* (Fig. 7a). Principal component (PC) analysis (PCA) across all 201 differentially methylated positions showed that n.4G>A carriers exhibited the strongest signal, although only ~25% of variance was captured by PC1 (Extended Data Fig. 8a and Supplementary Table 15). Separation of n.35A>G and biallelic carriers from controls became apparent only at higher PCs, reflecting the minor effect size. Excluding n.4G>A carriers improved visualization, with n.35A>G and biallelic variants separating partially from controls along PC2 (Extended Data Fig. 8b,c). Methylation profiles showed that n.4G>A carriers were largely hypomethylated, whereas n.35A>G carriers displayed modest hypermethylation (~2.5% Δ*β*) and biallelic carriers had heterogeneous patterns without consistent hypo- or hypermethylation (Fig. 7b). Cross-validation with a single four-class support vector machine (SVM) classifier confirmed the strongest signature for n.4G>A (sensitivity 100%), with lower performance for n.35A>G (83%) and biallelic (80%) variants and an overall specificity of 87% (Extended Data Fig. 8d). Overall, methylation differences between variant carriers and controls were less than 5% Δ*β*, near the technical detection limit, indicating subtle and heterogeneous effects across variants.

## Discussion

Although historically overlooked due to their high conservation and redundancy, variants in snRNAs are now recognized as principal contributors to genetic disorders. In this study, we analyzed all annotated snRNA genes, hypothesizing that some ‘pseudogenes’ may be functional disease genes. Our main finding was the identification of biallelic variants in *RNU2-2* as the cause of a frequent recessive NDD. Clinically, this recessive NDD closely resembles the recently described dominant *RNU2-2*–associated disorder caused by n.4G>A and n.35A>G^19,23^, but seems to be at least twice as common. The clinical phenotype associated with dominant or biallelic *RNU2-2* variants is characterized by neurodevelopmental delay, severe ID and epilepsy in 85% of cases, usually starting before 3 years of age. Excluding monoallelic de novo variants other than n.4G>A and n.35A>G, we estimate that dominant and recessive *RNU2-2* variants altogether account for at least ~0.35% of NDDs (Supplementary Table 7), similar to the prevalence of ReNU syndrome^16,18^. Interpreting *RNU2-2* variants is complicated by the relatively high and overlapping allele frequencies of pathogenic and benign variants. It is thus possible that a few biallelic variants reported in our study are nonpathogenic, whereas others not considered could contribute to disease or modify penetrance (like for example, n.99A>G). Compared to *RNU4-2*, which is associated mainly with dominant ReNU syndrome, *RNU2-2* shows lower constraint and greater tolerance to variation, consistent with its association with predominant recessive disorders. snRNA genes are hypermutable and saturated with variants^16,25^, probably explaining the discrepancy between their small size and the high frequency of the associated phenotypes.

The identification of *RNU2-2* biallelic variants as a common cause of recessive disorders was replicated independently by two groups^46–48^. Both other studies relied mainly on Genomics England data and report largely the same pathogenic variants, whereas our work is based on the PFMG and other independent cohorts, with no (or negligible) patient overlap. Despite this, the results are remarkably consistent, differing mainly in their interpretation. Unlike previous studies from the same authors, which reported de novo variants separately^19,23^, our data show that de novo variants in snRNA genes can underlie both recessive and dominant inheritance, reflecting their high mutability^25^. Therefore, a second pathogenic variant in trans should always be sought for when evaluating de novo variants in snRNA genes.

Jackson et al.^46^ suggest distinct mechanisms and clinical features for dominant versus recessive *RNU2-2* variants, whereas our detailed clinical analysis of >100 patients, combined with structural predictions of variant impacts, rather indicates a continuum between dominant and recessive disorders. *RNU2-2* exhibits a broad and complex spectrum of variant effects that blurs the lines between dominant and recessive inheritance. De novo variants near n.4G>A and n.35A>G are particularly hard to interpret in the absence of a second pathogenic allele. These variants are extremely rare or absent from population databases (that is, under stronger negative selection than other biallelic variants) and recur de novo across unsolved patients with NDD. Together, these findings suggest that certain heterozygous variants could contribute to dominant disease with variable penetrance, yet produce a classic recessive phenotype when paired with a second pathogenic allele in *trans*.

These observations must be interpreted in light of predicted variant effects on U2-2 snRNA and the presence of additional U2 copies, including canonical U2-1 (*RNU2-1*) paralogs. U2 contributes largely to defining splicing outcomes by selecting the intronic BS (consensus ‘YNYURAY’ in metazoans) and thereby the acceptor splice sites^49^. Before its integration within the spliceosome, U2 snRNAs undergo a maturation process that includes Sm-core assembly, nuclear reimport and protein recruitment to form the U2 snRNP^38,39,50–53^. The 3′ domain mediates structural interactions necessary for U2 snRNP biogenesis and spliceosome assembly^38,53,54^, such as SLIIa binding SF3B1^50,55^. In contrast, the 5′ domain contributes to spliceosome assembly, active site formation and branch-point recognition through highly conserved RNA–RNA interactions. During early spliceosome assembly, U2 initially probes the BS and forms a stable U2/BS branch helix^32^. This process requires the PRP5 ATPase, which remodels the BSL and enforces fidelity of BS recognition^40^. Formation of the branch helix may trigger SLI formation^55^, which is disrupted subsequently to allow tri-snRNP recruitment through creation of the U2/U6 helix II, to form the precatalytic spliceosome. Upon spliceosome activation, U2 forms two additional contiguous helices with U6 (Ia, Ib) that, along with helix II, flank and stabilize the RNA-based active site^56,57^. Variants in the Sm site or 3′ stem loops probably prevent U2 snRNP maturation and spliceosome incorporation. Mutant U2-2 RNAs that fail to mature properly behave like null alleles, similar to larger indels or whole-gene deletions. These null alleles are tolerated when heterozygous but pathogenic in homozygous or compound heterozygous states, highlighting the essential role of *RNU2-2* in human brain development. In contrast, variants in the U2-2 5′ region, spanning the U2/U6 helix II and BSL, are predicted to be incorporated into spliceosomes, although some may also alter U2-2 snRNA levels^58^. Recurrent variants n.4G>A and n.35A>G seem sufficient to reach the pathogenic threshold in the heterozygous state, whereas nearby variants are typically insufficient alone but can cause disease when paired with another variant in *trans*. This corresponds to a gradient-of-impact framework, where *RNU2-2* variants (1) can act dominantly on their own (n.4G>A; n.35A>G); (2) require a second nonfunctional allele to cause disease (one non-null allele, one null allele); (3) are both incorporated in spliceosomes, having additive effects (two non-null alleles) or (4) fail to enter the spliceosome entirely (two null alleles). Jackson et al. suggested that the U2-2:U2-1 ratio could serve as a biomarker for *RNU2-2* recessive disorders^46^. However, it is possible that this biomarker is valid only when at least one variant impairs U2-2 maturation.

Contrary to *RNU4-2*, *RNU2-2* variants exert a subtler effect on splicing in blood, causing primarily exon skipping for dominant variants and broader splicing alterations for recessive variants. These findings align with the lower expression of *RNU2-2* than *RNU2-1* in blood, which contrasts with its higher relative expression in the brain^19,23^, and is consistent with the primarily neurodevelopmental features of *RNU2-2*–associated disorders. Similarly, DNA methylation analyses in *RNU2-2* variant carriers revealed only modest, variant-specific changes. These results should be interpreted cautiously and validated in larger cohorts, as some differences may reflect stochastic variability rather than consistent, biologically meaningful episignatures, and may also mirror the variants’ diverse functional effects.

Despite the size of the PFMG cohort (>34,000 participants), we did not identify additional significant snRNA-disease associations. This probably reflects limited statistical power to detect variants in snRNA genes causing rarer disorders, as known disease associations with de novo variants in *RNU5B-1*^18,19^ or recessive variants in *RNU4-2*^21,22^, *RNU6ATAC*^59^ and *RNU12*^14,15^ did not reach significance. Increasing statistical power will require pooling international cohorts for robust genotype–phenotype association studies. Another important limitation of our study is the reliance on short-read genome sequencing, which cannot accurately resolve highly similar snRNA loci, including most U1 and U2 canonical genes, due to the presence of (nearly) identical copies contained in regions spanning >5–10 kb in sequence homology. The analysis of these regions thus requires long-read sequencing technologies.

In conclusion, our results support a continuum of dominant and recessive NDDs caused by *RNU2-2* variants, which altogether are nearly as prevalent as ReNU syndrome. They also underscore the challenges of interpreting variants, especially those occurring de novo, in these highly mutable loci, and suggest that additional snRNA-associated disorders remain to be discovered.

## Methods

### Inclusion and ethics statement

This study was conducted in accordance with the ethical standards and regulations of all participating countries. Written informed consent was obtained for all patients from their parents or legal guardians, with an additional consent form for families agreeing to the publication of photographs. For genetic analyses, patient samples were pseudonymized at each participating center. Information on the patients’ sex (but not gender) was extracted from clinical records. The promoters of this research study are Assistance Publique–Hôpitaux de Paris (AP–HP) for hospitals associated with the SeqOIA laboratory (project ID APHP241333) and Grenoble-Alpes University Hospital (CHU Grenoble-Alpes, research ID 19814188) for hospitals affiliated with the Auragen laboratory. Ethical approval was obtained from the University Hospital Essen (24-12010-BO) and the Comité Éthique et Scientifique pour les Recherches, les Études et les Évaluations dans le domaine de la Santé (CESREES; reference 21082803 Bis / 2038764). AP–HP has received an authorization from the Commission Nationale de l’Informatique et des Libertés (CNIL; reference HGTHGT/MFIMFI/AR2426865; request no. 924924336666) for data processing. Additional approvals were obtained from the ethics committee of CHU de Nantes (CCTIRS number 14.556) and from CPP Ouest V (File 06/15, Ref MESR DC 2017 2987; approval date 4 August 2015). For methylation analyses, DNA from patients and controls had been previously collected in a medical context for genetic testing, with written consent including authorization for research use of leftover material. Control samples consisted of participants without neurodevelopmental disorders, either unaffected relatives or persons tested presymptomatically for other conditions who were found not to carry pathogenic variants. DNA samples used for methylation profiling were stored within the genetics biobank of the CRBi, Rouen, France (collection DC 2008-711, authorization MCRBi/2024/02). The use of these samples was approved by the CERDE ethics committee of Rouen University Hospital (notification E2023-13). Researchers and clinicians from all contributing centers participated throughout the study, from design and implementation to data collection, analysis and manuscript preparation, and are listed as co-authors of this article.

### snRNA genes

Genes and pseudogenes corresponding to snRNAs were retrieved from Ensembl 113 BioMart^60,61^ by filtering on gene type ‘snRNA.’ Of the 2,094 genes, we excluded the ones placed in contigs, scaffolds or patches, keeping 1,901 genomic regions located in identifiable chromosomes. Of these, 1,741 genes are annotated as spliceosomal snRNAs. We downloaded through the UCSC Table Browser^62^: (1) the coordinates of the ENCODE Registry of candidate cCREs in the human genome^26^ and (2) the peaks of histone H3 acetylation of lysine 27 (H3K27ac) obtained for the H1 human embryonic stem cell line (H1-hESC)^27^. We annotated the 1,901 snRNAs by analyzing their genomic coordinates for overlaps with ENCODE cCREs and H3K27ac peaks. The *RNU* genes were further annotated for their hypermutability^25^. We kept for further analyses 200 putative functional snRNA genes that either overlap with ENCODE cCREs, are considered hypermutable or are approved by the HUGO Gene Nomenclature Committee (HGNC).

### Small RNA-seq data from embryonic brain tissues

We analyzed the expression level of the 1,901 snRNAs using small RNA-seq data generated from six human embryonic brain regions by the ENCODE Consortium^63^: diencephalon (GSE78292), temporal lobe (GSE78303), occipital lobe (GSE78298), frontal cortex (GSE78293), parietal lobe (GSE78299) and cerebellum (GSE78291). We downloaded 24 bigwig files from the ENCODE portal^26^ containing signals for ‘all reads’ and ‘unique reads,’ for plus and minus strands, from the default anisogenic replicate. For each genomic coordinate, we calculated the maximum normalized RNA-seq signal obtained from the bigwig files for the corresponding gene strand, as well as the number of covered bases. For plotting, we considered only snRNA genes for which at least 50% of the expected number of bases for the specific snRNA type were covered in at least one tissue when considering ‘all reads.’ The expected number of bases for each snRNA type were 164 (U1), 191 (U2), 141 (U4), 116 (U5), 107 (U6), 63 (U7), 127 (U4ATAC), 126 (U6ATAC), 134 (U11), 150 (U12) and 40 (other snRNAs).

### Patient cohorts and variant filtering

We first investigated monoallelic de novo and biallelic variants in snRNA genes in patients who underwent genome sequencing as part of the diagnostic process in France through Plan France Médecine Génomique 2025 (PFMG2025)^28^ on one of the two national clinical sequencing laboratories, SeqOIA (https://laboratoire-seqoia.fr/) and Auragen (https://www.auragen.fr/). Sequencing was performed in two subsets (Auragen and SeqOIA), with final inclusion dates in May and November 2025, respectively. gLEAVES, the system used for genome analysis on the SeqOIA platform, which is restricted to registered and accredited users, was used to investigate inheritance of variants and visualize bam files on Integrative Genomics Viewer (IGV). Because bioinformatics pipelines were run independently in each laboratory and differed slightly, each dataset was analyzed both separately and combined. The combined cohort included 34,329 patients with rare disorders (20,690 from SeqOIA and 13,639 from Auragen). Characteristics of the combined PFMG cohort and separated subcohorts and details of the filters appear in Extended Data Fig. 1 and Supplementary Fig. 4. Variants from 200 snRNA genes were extracted initially using the criteria: de novo or biallelic inheritance, and present in fewer than three homozygotes in gnomAD v.3 and fewer than five homozygotes in internal databases. Biallelic variants were defined as: (1) homozygous or hemizygous variants; (2) two heterozygous variants inherited from different parents for trios; (3) one inherited and one variant absent from the sequenced parent for duos or (4) one inherited and one de novo variant, with a *trans* configuration verified manually in IGV. Only variants with coverage at least ten in genes with median VAF ≥ 0.3 with less than 50% overlap with problematic regions in ‘Genome in a Bottle’ were considered for further analysis. Additional filtering for gene enrichment analyses included variants with gnomAD AC < 100 (‘rare variants’) and no gnomAD flags (for example, variants flagged as ‘LCR’). Two-sided Fisher’s test was used to test statistical enrichment in unsolved versus solved and in NDD versus non-NDD cases. Genes were kept for these analyses when the minimum number of patients carrying variants was sufficient to reach statistical significance (*P* < 0.05) in the cohort before multiple testing (unsolved versus solved: ten [PFMG, SeqOIA, Auragen]; NDD versus non-NDD: nine [PFMG, SeqOIA] or ten [Auragen]).

After the initial filtering, the criteria to select *RNU2-2* variants in the PFMG cohort were relaxed to broaden the inclusion of potentially pathogenic variants, restricting only on the homozygote frequency (<3 in gnomAD_v3 and <5 in internal databases) while removing restrictions on heterozygote frequency. We examined all cases with biallelic variants manually, and transitioned to the AoU database, which encompasses a larger cohort of genomes from healthy people, thereby providing greater power to assess population-level variation. Refined criteria were: AC < 50 for de novo variants and AC < 200 for biallelic variants and no homozygotes in AoU. Cases with de novo or P/LP variants were screened for a second variant in *trans*, revealing potentially pathogenic or modifier alleles with slightly higher population frequencies in a few families. Variant nomenclature was checked systematically with Variant Validator (https://variantvalidator.org/service/validate/).

To identify additional cases with *RNU2-*2 variants, we performed sequencing of *RNU2-2* and/or reanalyzed genome data in a cohort of 5,456 patients with NDDs (Extended Data Fig. 1 and Supplementary Table 7). Furthermore, cases with biallelic variants present in 24,958 genomes were identified using seqr^29^. This large international collaborative effort identified 34 additional cases: 13 patients with monoallelic variants (n.4G>A, *n* = 9; n.35A>G, *n* = 3; n.38A>G, *n* = 1) and 21 families with biallelic variants, originating from Germany (*n* = 9), France (*n* = 8), the US (*n* = 6), New Zealand (*n* = 3), Egypt (*n* = 3), Australia (*n* = 2), Denmark (*n* = 1), Turkey (*n* = 1) and Brazil (*n* = 1). None of these patients had been published previously, and we carefully verified the absence of duplicates among people carrying the same variant(s) by cross-checking year of birth and initials.

### Variant classification

We classified variants according to the American College of Medical Genetics and Genomics/Association for Molecular Pathology criteria using the recommendations of Ellingford et al.^64^. For de novo variants, PS2 was applied but downgraded in light of the hypermutable nature of snRNA genes: PS2_Supporting was applied for one de novo, PS2_Moderate when the same de novo variant was found in two to ten patients, and PS2 when the same de novo variant was found in more than ten patients (for n.4G>A and n.35A>G). PM2_Supporting was applied to variants absent or very rare in gnomAD v.3 (allele count <10) and AoU (allele count <50). PM1 was applied to variants located in functional domains U2/U6 helix II, SLI and BSL (n.1–n.45) and Sm binding site (n.98–n.107) and PM1_Supporting to variants in n.47-n.66 (SLIIa), n.68-n.84 (SLIIb), n.112–n.144 (SLIII) and n.147–n.184 (SLIV). PS4_Supporting was applied for variants present in two unrelated patients, PS4_Moderate when observed in three to four unrelated patients, and PS4 when present in five or more unrelated patients when the variant is rare (that is, PM2_sup is met). PS4 was downgraded for variants with allele count comprised between 11 and 50 in gnomAD v.3 and 51 to 250 in AoU (PS4_supp when observed in three to four unrelated patients, and PS4_Moderate when present in five or more unrelated patients). This criterion was not applied for more frequent variants. PP1 was applied if a variant cosegregated with disease in two or three affected family members, PP1_Moderate was applied when cosegregation was observed in five family members (affected or unaffected) or across several unrelated families. PM3 was assigned to variants detected in *trans* with a likely pathogenic or pathogenic variant.

We also annotated variants with MobiDeep available on Mobidetails website (https://mobidetails.chu-montpellier.fr)^65^. MobiDeep is a metascore for noncoding variants based on a multilayer perceptron using five features: REMM v.0.4, CADD 1.7, GPN-MSA and two conservation scores (Cactus 241-way vertebrates and PhyloP primates) to capture different evolutionary depths. Variants were stratified according to MobiDeep thresholds: <0.6 (neutral), >0.6 (probably deleterious) and >0.9684 (high-confidence deleterious).

### Sanger sequencing

Sanger targeted sequencing was performed to screen for variants in *RNU2-2* and/or to perform segregation analysis. PCR amplification of *RNU2-2* was performed using the HotStarTaq Master Mix Kit (Qiagen, cat. no. 203445) with the following primers: F: 5′- CAAACACGCGTCATTCAACACAC-3′; R: 5′- ATAACTGGTTGGAAGATGGGAAGG-3′ (designed using primer3), according to the manufacturer’s instructions. Forward and reverse sequencing reactions were performed using the BrilliantDye Terminator v.1.1 Cycle Sequencing Kit (Nimagen; cat. no. BRD1-1000) or the BigDye Terminator v.3.1 Sequencing Kit (Life Technologies, cat. no. 4337457). ExoSAP-Purified sequencing products (ExoSAP-IT, Applied Biosystem, cat. no. 78205) were run on Pop-7 polymer (Life Technologies, cat. no. 4335615) using an ABI 3730 or 3730XL automated sequencer (Applied Biosystems). Sequences were analyzed using Geneious Prime 2019 (Biomatters Ltd.) or Seqscape v.2.6 software (Applied Biosystems).

### Clinical analyses

Clinical data were collected retrospectively from the referring physician using an anonymized Excel sheet, as published previously^18^. We collected categorical data on 66 clinical features from 112 patients. For clustering and statistical comparisons, we used 60 features from 87 index patients, except in the analysis restricted to patients carrying biallelic variants, where 59 features from 56 patients were included since the remaining features showed no variation among patients. Data were converted to a 0–1 scale, with 0 representing a more favorable phenotype presentation and 1 a more severe phenotype. Hierarchical clustering was performed using pheatmap R package, performing *Z* score scaling for each row (across different patients), and ward.D2 clustering method keeping missing values. PCA was generated after replacing missing data by 0 and performing variable scaling. Microcephaly was defined as head circumference measurements less than the third percentile. We used charts established by Fenton et al.^66^ to calculate head circumference percentile at birth and define congenital microcephaly. Fisher’s tests (two-sided; 2 × 2, 2 × 3, 2 × 4 or 2 × 5 contingency tables) adjusted for multiple comparisons using Bonferroni correction were used to compare clinical features from patients with different variant types (n.4G>A versus n.35A>G: 39 (index only) or 37 (including siblings) clinical features; de novo versus biallelic variants: 48 (index only) or 50 (including siblings) clinical features). Clinical features were kept for the statistical analysis when the minimum number of patients with gathered information was sufficient to reach statistical significance (*P* < 0.05) before multiple testing.

### Prediction of variant impact

Structural analysis of variants and corresponding figures were performed using the PyMol v.3.0.0 visualization software^67^ on published coordinates of the human U2 snRNP structures: PDB 7Q3L ref. ^30^ for the BSL structure and 5XJC ref. ^31^ for the representation of the rest.

### RNA sequencing

RNA-seq experiments were conducted following a similar protocol and workflow previously described for *RNU4-2*^18^. Briefly, PBMCs were isolated from 2–4 ml of EDTA blood and cultured in lymphocyte-stimulating medium for 48–72 h. RNA was extracted and stranded RNA-seq libraries prepared from 100 ng total RNA using the SureSelect XT-HS2 kit (Human All Exon V8 capture probes; cat. no. G9774C), followed by sequencing on an Illumina NextSeq 550 to obtain ~25–30 million paired-end reads per sample. Reads were aligned to GRCh38 with STAR v.2.7.11a. Quality control was performed with FastQC v.0.11.3 and Fastp v.0.23.4. Cell type composition was estimated using CIBERSORTx v.1.0 with the LM22 signature matrix. For splicing analysis, we focused on 19 people carrying either dominant (*n*.4G>A, *n* = 5; *n*.35A>G, *n* = 5) or biallelic (*n* = 9) *RNU2-2* variants that we compared to 49 controls (probands without *RNU2-2* variants). rMATS-turbo v.4.3.0 was used to detect alternative splicing events with the following parameters: -t paired –anchorLength 1 –libType fr-firststrand –novelSS –variable-read-length –allow-clipping. rMATS outputs were filtered for mean coverage >10 and ΔPSI > 0.05. For each splicing event, we fitted a linear regression model adjusting for age, sex and estimated cell composition using ordinary least squares with statsmodels (v.0.14.5) in Python (v.3.12.2). *P* values were obtained for association with affected status; significant splicing events were defined as those with *P* value < 0.01, retaining only the most significant event per gene. To visualize results, the linear regression model was trained on the 49 control samples and applied to both controls and affected patients; residual PSI values were then used for PCA using sklearn (v.1.7.2) and clustermaps using seaborn (v.0.13.2) for the splicing categories: SE, 5′SS, 3′SS and RI. Sashimi plot was performed with rmats2sashimi.

### DNA methylation study

DNA methylation analysis was performed as described previously^18^. Genomic DNA was extracted from whole blood and bisulfite converted. DNA methylation profiles were obtained using Infinium MethylationEPIC v.2.0 BeadChips (Illumina, cat. no. 20087708) following the manufacturer’s protocol. Patients and controls were balanced across 50 arrays and array rows to reduce technical bias and matched for age at sampling. Arrays were processed at the ASGARD-Rouen genomic platform (Rouen University Hospital, University of Rouen) on an Illumina NextSeq 550 scanner. Raw IDAT files were normalized using Meffil R package, including all 50 arrays to estimate variability within and across arrays. Functional normalization was performed with random effect adjustment for array and sentrix row and fixed effect adjustment for the first two PCs, generating *β*-values.

An epigenome-wide association study was conducted using normalized *β* values from controls and patients with P or LP dominant or biallelic *RNU2-2* variants. Multiple linear regression was applied per CpG to assess methylation differences, adjusting for age, sex and predicted blood cell composition. To account for potential heterogeneity, two variant-specific coefficients (dominant n.35A>G and biallelic recessive) were added alongside the main case–control effect, with n.4G>A as the reference. Regression coefficients and *P* values were extracted for all three variables. CpGs were considered significant if *P* < 1 × 10^−5^ and absolute effect size >0.05 for any coefficient. Significant CpGs were classified based on association patterns. Probes with only a main differential effect were labeled ‘common to all variants.’ Probes significant only for n.35A>G or biallelic coefficients were labeled ‘specific to n.35A>G’ or ‘specific to biallelic recessive carriers,’ respectively. Probes significant for both main and variant-specific coefficients corresponded to n.4G>A-specific probes after visual confirmation of effect directions.

Adjusted methylation levels were visualized using PCA and heatmaps (pheatmap, Euclidean distance, Ward aggregation method). Baseline methylation models were fitted on controls, correcting normalized *β* value for age, sex and blood composition. CpGs were grouped by association patterns (common, n.35A>G-specific, biallelic-specific, n.4G>A-specific) and mean adjusted methylation per sample was calculated. Raincloud plots were generated (ggplot2, ggdist) combining half-violin distributions, boxplots and jittered points colored by group. Pairwise comparisons were performed using two-sided Wilcoxon rank-sum tests (ggpubr), with displaying significant *P* values only.

Predictive performance of the CpG signature was assessed using a multiclass linear kernel SVM (e1071) with four outcomes: Control, Dominant n.4G>A, Dominant n.35A>G, Recessive biallelic. Three-block cross-validation was applied within each variant group, training on two blocks and testing on the third, ensuring each sample was predicted once. For each cross-validation iteration, a new differential methylation analysis was performed and the SVM classifier was trained on the resulting CpG signature. Predicted labels and class probabilities were obtained, and sensitivity and specificity per class were calculated with 95% confidence intervals (Wilson method). Probabilities were averaged and visualized to illustrate class separation and model confidence.

### Reporting summary

Further information on research design is available in the Nature Portfolio Reporting Summary linked to this article.

## Online content

Any methods, additional references, Nature Portfolio reporting summaries, source data, extended data, supplementary information, acknowledgements, peer review information; details of author contributions and competing interests; and statements of data and code availability are available at 10.1038/s41588-026-02547-5.

## Supplementary information

Supplementary Information Supplementary Notes and Figs. 1–7.

Reporting Summary

Peer Review File

Supplementary Table 1 Supplementary Tables 1–15.

## Data Availability

Variant details have been submitted to ClinVar (SUB15855213). RNA-seq and methylation data have been deposited in the European Genome-phenome Archive (EGA, http://www.ebi.ac.uk/ega), hosted by the EBI. RNA-seq data are available under the Study Accession Number EGAS50000001410. Methylation data are accessible under the study accession EGAS00001008070. Both are subject to a Data Processing Agreement, and access requests will be reviewed by a Data Access Committee to ensure compliance with ethical and legal standards. Due to ethical considerations, individual genome data cannot be made publicly available. Controlled access is required to safeguard participant privacy and to comply with data protection regulations, including the GDPR in Europe. Access to genome data from the PFMG2025 cohort is governed by French data protection laws and is only possible through the Collecteur Analyseur de Données (CAD). More details can be found on the PFMG2025 website at https://pfmg2025.fr/le-plan/collecteur-analyseur-de-donnees-cad/. The coordinates of the ENCODE Registry of cCREs in the human genome^26^ and bigwig files concerning the peaks of histone H3 acetylation of lysine 27 (H3K27ac) obtained for the H1 human embryonic stem cell line (H1-hESC)^27^ were downloaded through the UCSC Table Browser^62^. Bigwig files concerning small RNA-seq data from six human embryonic brain regions were downloaded from the ENCODE portal^68^ (https://www.encodeproject.org/) with the following identifiers: ENCFF013RLG, ENCFF029RIV, ENCFF034QAV, ENCFF197SSE, ENCFF221KEN, ENCFF343ZBS, ENCFF405QIN, ENCFF532SOY, ENCFF654ONK, ENCFF738LDD, ENCFF870FMA, ENCFF887TOS, ENCFF106ESQ, ENCFF222WBQ, ENCFF250WEA, ENCFF254UEQ, ENCFF319GRF, ENCFF425WUZ, ENCFF443ONL, ENCFF820JTT, ENCFF897IWP, ENCFF915WAC, ENCFF946YVE and ENCFF965GHD.

## Code availability

No custom code was developed for this study. All analyses were performed using existing, publicly available pipelines and software tools listed in the Methods and Reporting Summary.

## References

[1] Wilkinson, M. E., Charenton, C. & Nagai, K. RNA splicing by the spliceosome. *Annu. Rev. Biochem.* **89**, 359–388 (2020). 31794245 10.1146/annurev-biochem-091719-064225

[2] Charenton, C., Wilkinson, M. E. & Nagai, K. Mechanism of 5′ splice site transfer for human spliceosome activation. *Science* **364**, 362–367 (2019). 30975767 10.1126/science.aax3289 PMC6525098

[3] Moyer, D. C., Larue, G. E., Hershberger, C. E., Roy, S. W. & Padgett, R. A. Comprehensive database and evolutionary dynamics of U12-type introns. *Nucleic Acids Res.* **48**, 7066–7078 (2020). 32484558 10.1093/nar/gkaa464 PMC7367187

[4] Mabin, J. W., Lewis, P. W., Brow, D. A. & Dvinge, H. Human spliceosomal snRNA sequence variants generate variant spliceosomes. *RNA* **27**, 1186–1203 (2021). 34234030 10.1261/rna.078768.121 PMC8457000

[5] Guiro, J. & O’Reilly, D. Insights into the U1 small nuclear ribonucleoprotein complex superfamily. *Wiley Interdiscip. Rev. RNA* **6**, 79–92 (2015). 25263988 10.1002/wrna.1257

[6] Bernstein, L. B., Manser, T. & Weiner, A. M. Human U1 small nuclear RNA genes: extensive conservation of flanking sequences suggests cycles of gene amplification and transposition. *Mol. Cell. Biol.* **5**, 2159–2171 (1985). 3837185 10.1128/mcb.5.9.2159-2171.1985 PMC366940

[7] Lindgren, V., Bernstein, L. B., Weiner, A. M. & Francke, U. Human U1 small nuclear RNA pseudogenes do not map to the site of the U1 genes in 1p36 but are clustered in 1q12-q22. *Mol. Cell. Biol.* **5**, 2172–2180 (1985). 3837186 10.1128/mcb.5.9.2172-2180.1985 PMC366941

[8] Tessereau, C. et al. Direct visualization of the highly polymorphic *RNU2* locus in proximity to the *BRCA1* gene. *PLoS ONE* **8**, e76054 (2013). 24146815 10.1371/journal.pone.0076054 PMC3795722

[9] Duker, A. et al. RNU4atac-opathy. in *GeneReviews* (eds Adam, M. P. et al.) https://www.ncbi.nlm.nih.gov/books/NBK589232/ (Univ. of Washington, 2023). 36795902

[10] Edery, P. et al. Association of TALS developmental disorder with defect in minor splicing component U4atac snRNA. *Science* **332**, 240–243 (2011). 21474761 10.1126/science.1202205

[11] He, H. et al. Mutations in U4atac snRNA, a component of the minor spliceosome, in the developmental disorder MOPD I. *Science* **332**, 238–240 (2011). 21474760 10.1126/science.1200587 PMC3380448

[12] Merico, D. et al. Compound heterozygous mutations in the noncoding *RNU4ATAC* cause Roifman syndrome by disrupting minor intron splicing. *Nat. Commun.* **6**, 8718 (2015). 26522830 10.1038/ncomms9718 PMC4667643

[13] Farach, L. S. et al. The expanding phenotype of *RNU4ATAC* pathogenic variants to Lowry Wood syndrome. *Am. J. Med. Genet. A* **176**, 465–469 (2018). 29265708 10.1002/ajmg.a.38581 PMC6774248

[14] Elsaid, M. F. et al. Mutation in noncoding RNA *RNU12* causes early onset cerebellar ataxia. *Ann. Neurol.* **81**, 68–78 (2017). 27863452 10.1002/ana.24826

[15] Xing, C. et al. Biallelic variants in *RNU12* cause CDAGS syndrome. *Hum. Mutat.* **42**, 1042–1052 (2021). 34085356 10.1002/humu.24239

[16] Chen, Y. et al. De novo variants in the RNU4-2 snRNA cause a frequent neurodevelopmental syndrome. *Nature* **632**, 832–840 (2024). 38991538 10.1038/s41586-024-07773-7 PMC11338827

[17] Greene, D. et al. Mutations in the U4 snRNA gene *RNU4-2* cause one of the most prevalent monogenic neurodevelopmental disorders. *Nat. Med.* **30**, 2165–2169 (2024). 38821540 10.1038/s41591-024-03085-5 PMC11333284

[18] Nava, C. et al. Dominant variants in major spliceosome U4 and U5 small nuclear RNA genes cause neurodevelopmental disorders through splicing disruption. *Nat. Genet.* **57**, 1374–1388 (2025). 40379786 10.1038/s41588-025-02184-4 PMC12165858

[19] Jackson, A. et al. Analysis of R-loop forming regions identifies *RNU2-2* and *RNU5B-1* as neurodevelopmental disorder genes. *Nat. Genet.* **57**, 1362–1366 (2025). 40442284 10.1038/s41588-025-02209-y PMC12165836

[20] Quinodoz, M. et al. De novo and inherited dominant variants in U4 and U6 snRNA genes cause retinitis pigmentosa. *Nat. Genet.* **58**, 169–179 (2026). 41513982 10.1038/s41588-025-02451-4 PMC12807869

[21] De Jonghe, J. et al. Saturation editing of *RNU4-2* reveals distinct dominant and recessive disorders. *Nature*10.1038/s41586-026-10334-9 (2026). 10.1038/s41586-026-10334-9 PMC13253345 41951737

[22] Rius, R. et al. Biallelic variants in the non-coding RNA gene *RNU4-2* cause a recessive neurodevelopmental syndrome with distinct white matter changes. *Nat. Genet*. 10.1038/s41588-026-02554-6 (2026). 10.1038/s41588-026-02554-6 PMC13083247 41951959

[23] Greene, D. et al. Mutations in the small nuclear RNA gene RNU2-2 cause a severe neurodevelopmental disorder with prominent epilepsy. *Nat. Genet.* **57**, 1367–1373 (2025). 40210679 10.1038/s41588-025-02159-5 PMC12165851

[24] Bousquets-Munoz, P. et al. PanCancer analysis of somatic mutations in repetitive regions reveals recurrent mutations in snRNA U2. *NPJ Genom. Med.* **7**, 19 (2022). 35288589 10.1038/s41525-022-00292-2 PMC8921233

[25] Seplyarskiy, V. et al. A mutation rate model at the basepair resolution identifies the mutagenic effect of polymerase III transcription. *Nat. Genet.* **55**, 2235–2242 (2023). 38036792 10.1038/s41588-023-01562-0 PMC11348951

[26] ENCODE Project Consortium et al. Expanded encyclopaedias of DNA elements in the human and mouse genomes. *Nature* **583**, 699–710 (2020). 32728249 10.1038/s41586-020-2493-4 PMC7410828

[27] ENCODE Project Consortium An integrated encyclopedia of DNA elements in the human genome. *Nature* **489**, 57–74 (2012). 22955616 10.1038/nature11247 PMC3439153

[28] PFMG2025 contributors. *PFMG2025*–integrating genomic medicine into the national healthcare system in France. *Lancet Reg. Health Eur.* **50**, 101183 (2025). 40093400 10.1016/j.lanepe.2024.101183 PMC11910791

[29] Pais, L. S. et al. seqr: a web-based analysis and collaboration tool for rare disease genomics. *Hum. Mutat.* **43**, 698–707 (2022). 35266241 10.1002/humu.24366 PMC9903206

[30] Tholen, J., Razew, M., Weis, F. & Galej, W. P. Structural basis of branch site recognition by the human spliceosome. *Science* **375**, 50–57 (2022). 34822310 10.1126/science.abm4245 PMC7614990

[31] Zhang, X. et al. An atomic structure of the human spliceosome. *Cell* **169**, 918–929 (2017). 28502770 10.1016/j.cell.2017.04.033

[32] Perriman, R. & Ares, M. Jr. Invariant U2 snRNA nucleotides form a stem loop to recognize the intron early in splicing. *Mol. Cell* **38**, 416–427 (2010). 20471947 10.1016/j.molcel.2010.02.036 PMC2872779

[33] Wu, J. & Manley, J. L. Multiple functional domains of human U2 small nuclear RNA: strengthening conserved stem I can block splicing. *Mol. Cell. Biol.* **12**, 5464–5473 (1992). 1448079 10.1128/mcb.12.12.5464-5473.1992 PMC360484

[34] Ryan, D. E. & Abelson, J. The conserved central domain of yeast U6 snRNA: importance of U2-U6 helix Ia in spliceosome assembly. *RNA* **8**, 997–1010 (2002). 12212854 10.1017/s1355838202025013 PMC1370321

[35] Gozani, O., Potashkin, J. & Reed, R. A potential role for U2AF-SAP 155 interactions in recruiting U2 snRNP to the branch site. *Mol. Cell. Biol.* **18**, 4752–4760 (1998). 9671485 10.1128/mcb.18.8.4752 PMC109061

[36] Stallings, S. C. & Moore, P. B. The structure of an essential splicing element: stem loop IIa from yeast U2 snRNA. *Structure* **5**, 1173–1185 (1997). 9331416 10.1016/s0969-2126(97)00268-2

[37] Branlant, C. et al. U2 RNA shares a structural domain with U1, U4, and U5 RNAs. *EMBO J.* **1**, 1259–1265 (1982). 6202507 10.1002/j.1460-2075.1982.tb00022.x PMC553198

[38] Dybkov, O. et al. U2 snRNA-protein contacts in purified human 17S U2 snRNPs and in spliceosomal A and B complexes. *Mol. Cell. Biol.* **26**, 2803–2816 (2006). 16537922 10.1128/MCB.26.7.2803-2816.2006 PMC1430325

[39] Raker, V. A., Plessel, G. & Luhrmann, R. The snRNP core assembly pathway: identification of stable core protein heteromeric complexes and an snRNP subcore particle in vitro. *EMBO J.* **15**, 2256–2269 (1996). 8641291 PMC450151

[40] Zhang, X. et al. Structural insights into branch site proofreading by human spliceosome. *Nat. Struct. Mol. Biol.* **31**, 835–845 (2024). 38196034 10.1038/s41594-023-01188-0

[41] Donmez, G., Hartmuth, K. & Luhrmann, R. Modified nucleotides at the 5’ end of human U2 snRNA are required for spliceosomal E-complex formation. *RNA* **10**, 1925–1933 (2004). 15525712 10.1261/rna.7186504 PMC1370681

[42] Boesler, C. et al. A spliceosome intermediate with loosely associated tri-snRNP accumulates in the absence of Prp28 ATPase activity. *Nat. Commun.* **7**, 11997 (2016). 27377154 10.1038/ncomms11997 PMC4935976

[43] Roy, A., Panigrahi, S., Bhattacharyya, M. & Bhattacharyya, D. Structure, stability, and dynamics of canonical and noncanonical base pairs: quantum chemical studies. *J. Phys. Chem. B* **112**, 3786–3796 (2008). 18318519 10.1021/jp076921e

[44] Wu, G. et al. Pseudouridines in U2 snRNA stimulate the ATPase activity of Prp5 during spliceosome assembly. *EMBO J.* **35**, 654–667 (2016). 26873591 10.15252/embj.201593113 PMC4801943

[45] Zhuang, Y. & Weiner, A. M. A compensatory base change in human U2 snRNA can suppress a branch site mutation. *Genes Dev.* **3**, 1545–1552 (1989). 2612904 10.1101/gad.3.10.1545

[46] Jackson, A. et al. Biallelic variants in *RNU2-2* cause a remarkably frequent developmental epileptic encephalopathy. Preprint at *medRxiv*10.1101/2025.09.02.25334957 (2025). 10.1038/s41588-026-02551-9 PMC13083258 41912933

[47] Greene, D. et al. Biallelic variants in *RNU2-2* cause the most prevalent known recessive neurodevelopmental disorder. Preprint at *medRxiv*10.1101/2025.08.26.25334179 (2025). 10.1038/s41588-026-02539-5 PMC13083235 41912932

[48] Leitao, E. et al. Systematic analysis of snRNA genes reveals frequent *RNU2-2* variants in dominant and recessive developmental and epileptic encephalopathies. Preprint at *medRxiv*10.1101/2025.09.02.25334923 (2025). 10.1038/s41588-026-02547-5 PMC13083260 41912934

[49] Zhuang, Y. & Weiner, A. M. The conserved dinucleotide AG of the 3′ splice site may be recognized twice during in vitro splicing of mammalian mRNA precursors. *Gene* **90**, 263–269 (1990). 2401404 10.1016/0378-1119(90)90189-x

[50] Zhang, Z. et al. Molecular architecture of the human 17S U2 snRNP. *Nature* **583**, 310–313 (2020). 32494006 10.1038/s41586-020-2344-3

[51] Will, C. L. & Luhrmann, R. Spliceosomal UsnRNP biogenesis, structure and function. *Curr. Opin. Cell Biol.* **13**, 290–301 (2001). 11343899 10.1016/s0955-0674(00)00211-8

[52] Guthrie, C. & Patterson, B. Spliceosomal snRNAs. *Annu. Rev. Genet.* **22**, 387–419 (1988). 2977088 10.1146/annurev.ge.22.120188.002131

[53] Plaschka, C., Lin, P. C., Charenton, C. & Nagai, K. Prespliceosome structure provides insights into spliceosome assembly and regulation. *Nature* **559**, 419–422 (2018). 29995849 10.1038/s41586-018-0323-8 PMC6141012

[54] Pan, Z. Q. & Prives, C. U2 snRNA sequences that bind U2-specific proteins are dispensable for the function of U2 snRNP in splicing. *Genes Dev.* **3**, 1887–1898 (1989). 2559872 10.1101/gad.3.12a.1887

[55] Zhang, Z. et al. Structural insights into how Prp5 proofreads the pre-mRNA branch site. *Nature* **596**, 296–300 (2021). 34349264 10.1038/s41586-021-03789-5 PMC8357632

[56] Burke, J. E., Sashital, D. G., Zuo, X., Wang, Y. X. & Butcher, S. E. Structure of the yeast U2/U6 snRNA complex. *RNA* **18**, 673–683 (2012). 22328579 10.1261/rna.031138.111 PMC3312555

[57] Zhang, X. et al. Structure of the human activated spliceosome in three conformational states. *Cell Res.* **28**, 307–322 (2018). 29360106 10.1038/cr.2018.14 PMC5835773

[58] Stevers, M. B., Katzman, S. & Jurica, M. S. The human branchpoint-interacting stem loop sequence and structure regulates U2 snRNA expression, branchpoint recognition, and transcriptome. Preprint at *bioRxiv*10.1101/2025.08.31.673180 (2025). 10.1093/nar/gkag245 PMC13019295 41885209

[59] Arriaga, T. M. et al. Transcriptome-wide outlier approach identifies individuals with minor spliceopathies. *Am. J. Hum. Genet.* **112**, 2458–2475 (2025). 40975062 10.1016/j.ajhg.2025.08.018 PMC12696491

[60] Kinsella, R. J. et al. Ensembl BioMarts: a hub for data retrieval across taxonomic space. *Database (Oxford)* **2011**, bar030 (2011). 21785142 10.1093/database/bar030 PMC3170168

[61] Harrison, P. W. et al. Ensembl 2024. *Nucleic Acids Res.* **52**, D891–D899 (2024). 37953337 10.1093/nar/gkad1049 PMC10767893

[62] Perez, G. et al. The UCSC Genome Browser database: 2025 update. *Nucleic Acids Res.* **53**, D1243–D1249 (2025). 39460617 10.1093/nar/gkae974 PMC11701590

[63] Rahmanian, S. et al. Dynamics of microRNA expression during mouse prenatal development. *Genome Res.* **29**, 1900–1909 (2019). 31645363 10.1101/gr.248997.119 PMC6836743

[64] Ellingford, J. M. et al. Recommendations for clinical interpretation of variants found in non-coding regions of the genome. *Genome Med.* **14**, 73 (2022). 35850704 10.1186/s13073-022-01073-3 PMC9295495

[65] Baux, D. et al. MobiDetails: online DNA variants interpretation. *Eur. J. Hum. Genet.* **29**, 356–360 (2021). 33161418 10.1038/s41431-020-00755-z PMC7868358

[66] Fenton, T. R. & Kim, J. H. A systematic review and meta-analysis to revise the Fenton growth chart for preterm infants. *BMC Pediatr.* **13**, 59 (2013). 23601190 10.1186/1471-2431-13-59 PMC3637477

[67] Schrödinger, L. & DeLano, W. PyMOL (OriginLab Corporation, 2020).

[68] Sloan, C. A. et al. ENCODE data at the ENCODE portal. *Nucleic Acids Res.* **44**, D726–D732 (2016). 26527727 10.1093/nar/gkv1160 PMC4702836

