# Supplementary material for: Systematic analysis of snRNA genes reveals frequent RNU2-2 variants in dominant and recessive developmental and epileptic encephalopathies
Source: Nat Genet. 2026 Mar 30;58(4):782–97. doi: 10.1038/s41588-026-02547-5 (PMC13083260; doi:10.1038/s41588-026-02547-5)
Supplement: Supplementary file 1 — Supplementary Notes and Figs. 1–7. [file 41588_2026_2547_MOESM1_ESM.pdf]

nature genetics

Supplementary information

https://doi.org/10.1038/s41588-026-02547-5

Systematic analysis of snRNA genes reveals frequent *RNU2-2* variants in dominant and recessive developmental and epileptic encephalopathies

In the format provided by the authors and unedited

Table of Contents

Table of Contents 1

Supplementary Notes 2

Analysis of *de novo* and biallelic variants in putatively functional snRNA genes in the PFMG cohort 2

Analysis of *RNU2-2* variants in the PFMG and other cohorts 2

References 3

Supplementary Figures 4

Supplementary Fig. 1. Variant distribution across 200 putatively functional genes in the Auragen and SeqOIA PFMG subcohorts 4

Supplementary Fig. 2. Variant allele fraction (VAF) distributions of snRNA variants in SeqOIA and Auragen subcohorts 5

Supplementary Fig. 3. gnomAD allele count (AC) distribution of snRNA variants variants in SeqOIA and Auragen subcohorts 6

Supplementary Fig. 4. Details of the PFMG subcohorts and variant filtering strategy 7

Supplementary Fig. 5. Comparison of rare variant burden in snRNA genes in unsolved versus solved cases separating the PFMG cohort into SeqOIA and Auragen subparts 8

Supplementary Fig. 6. Comparison of rare variant burden in snRNA genes in NDD versus non-NDD cases separating the PFMG cohort into SeqOIA and Auragen subparts 9

Supplementary Fig. 7. Pearson's correlation between gnomAD v3 and All of Us allele counts for *RNU2-2* variants 10

Supplementary Notes

Analysis of *de novo* and biallelic variants in putatively functional snRNA genes in the PFMG cohort

We analysed variants in the 200 potentially functional snRNA genes in the Plan France Médecine Génomique 2025 (PFMG) cohort.[1] At the time of the study, this cohort consisted in short-read genome data from 34,329 patients with rare disorders (22,775 with NDDs). We retrieved variants compatible with monogenic inheritance in these genes: 1) dominant *de novo* in trios with unaffected parents; and 2) biallelic variants (at least two variants, each inherited by one parent when parents were available; at least two variants, one inherited and the other absent form the only parent sequenced for duos; or one *de novo* and one inherited variant).

A total of 11,055 variants (5,196 *de novo* and 8,784 biallelic) in 121 genes across a total of 5,567 patients were identified: 9,109 variants (4,685 *de novo* and 7,201 biallelic) in 105 genes in SeqOIA and 1,946 variants (511 *de novo* and 1,583 biallelic) in 75 genes in Auragen (Extended Data Fig. 1-2; Supplementary Fig. 1). Analysis of variant allele fractions (VAF) for variants covered by ≥10 reads showed that many snRNA gene variants fall in the low range of 0.1-0.3. This pattern, seen in both sub-cohorts, reflects the limitation of short-read sequencing in distinguishing identical or highly similar snRNA copies. Recurrent low VAFs were observed in *RNU1-1, RNU1-2, RNU2-1,* other closely related U1 and U2 copies, and several U6 genes, consistent with their high sequence identity (Extended Data Fig. 3; Supplementary Fig. 2). To address this, we excluded 46 genes: 6 with predominantly low-VAF variants, 31 overlapping >50% with the GIAB Problematic Regions UCSC track, and 9 with both features. Following this filtering, we retained a total of 843 high-quality variants in 66 genes, including 330 *de novo* and 551 biallelic variants, in a total of 616 patients in the entire PFMG cohort (Extended Data Fig. 1).

Analysis of *RNU2-2* variants in the PFMG and other cohorts

After the discovery phase, we used relaxed criteria to identify possibly pathogenic variants in *RNU2-2* with less stringent criteria. We listed all *RNU2-2* variants compatible with *de novo* or biallelic inheritance including variants only when present in the homozygous state fewer than 3 times in gnomAD v3 or fewer than 5 times in internal databases (see Methods). We then transitioned to the *All of Us* database, which contain a larger number of genomes from unaffected individuals to apply the 'rare' criteria as follows: AC <50 for *de novo* variants and <200 for biallelic variants.

Using these criteria, 42 unrelated patients had a rare *de novo RNU2-2* variant in the PFMG cohort (Supplementary Table 6). Of those, 21 probands had either n.4G>A (*n*=11) or n.35A>G (*n*=10).

In addition, n.4G>A variant was present in a patient for whom parents were not available. A single individual with n.4G>A had the n.80A>G in trans. The remaining 21 patients had other *de novo* variants: n.5C>A, n.6T>C (*n*=2), n.7_8insA (*n*=2), n.8C>T, n.21C>G, n.31G>A, n.37T>G (*n*=2), n.40C>G, n.40C>T (*n*=3), n.62T>G, n.63_64insC, n.101T>A, n.109A>T, n.129_139del, n.143_167del, and n.150T>G (Fig. 3). In 7/21 patients (including with n.5C>A, n.6T>C, n.7_8insA, n.31G>A, 37T>G (*n*=2), and n.40C>T, a second rare variant in *trans* was identified. In addition to the seven cases, 45 index cases (42 NDD and 3 non-NDDs) had rare biallelic variants in the PFMG cohort.

Genome reanalysis or targeted analysis of *RNU2-2*, performed in different laboratories identified 13 additional individuals with either pathogenic or *de novo* monoallelic variants and 21 additional families with biallelic variants (Extended Data Fig. 3; Supplementary Table 8).

In total, 34 index cases and a monozygotic twin had dominant pathogenic variants (n.4G>A and n.35A>G); 15 patients had a single *de novo* heterozygous variant other than n.4G>A and n.35A>G, and 91 patients from 73 unrelated families with biallelic variants. Biallelic variants segregated with the disorder in 16 families, and none of the tested unaffected siblings carried both variants (Fig. 4a; Extended Data Fig. 6).

We identified 92 distinct variants that we classified according to ACMG criteria (Supplementary Table 8). Twenty-two variants other than n.4G>A and n.35A>G were recurrently found in unrelated families (n.45C>T, *n*=11; n.100T>C, *n*=6; n.20G>A, n.40C>G, n.40C>T, n.107_118del, *n*=5 each; n.25G>A, n.28C>G, n.61C>G, n.104T>C, n.128C>T, *n*=3 each; n.6T>C, n.7_8insA, n.13C>T, n.31G>A, n.32T>G, n.37T>G, n.101T>A, n.104T>G, n.113G>A, n.116_127del, n.181G>C, *n*=2 each). Taking variants published by Jackson et al. [2] and Greene et al. [3] into account, twelve variants were classified as LP: n.6T>C; n.8C>T; n.19G>A; n.20G>A; n.28C>G; n.31G>A; n.32T>G; n.61C>G; n.100T>C; n.100T>G; n.104T>G; n.107_118del).

References

1. PFMG2025 contributors. PFMG2025–integrating genomic medicine into the national healthcare system in France. *Lancet Reg Health Eur.* **50**(2025).
2. Jackson, A. *et al.* Biallelic variants in RNU2-2 cause a remarkably frequent developmental epileptic encephalopathy. *medRxiv*, 2025.09.02.25334957 (2025).
3. Greene, D. *et al.* Biallelic variants in RNU2-2 cause the most prevalent known recessive neurodevelopmental disorder. *medRxiv* (2025).

Supplementary Figures

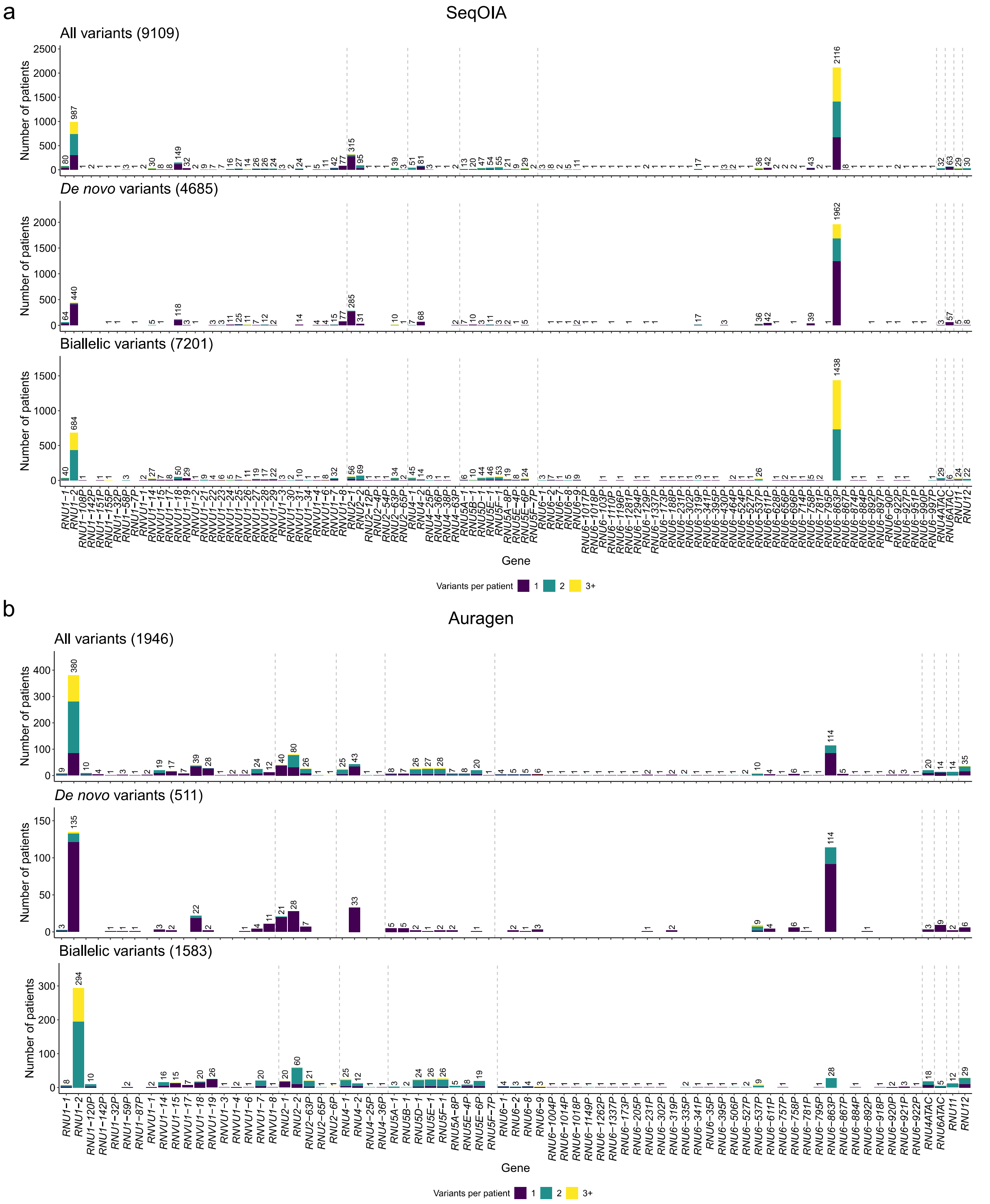

Supplementary Fig. 1. Variant distribution across 200 putatively functional genes in the Auragen and SeqOIA PFMG subcohorts. a, SeqOIA. Variants were found in 105 genes. **b,** Auragen. Variants were found in 75 genes.

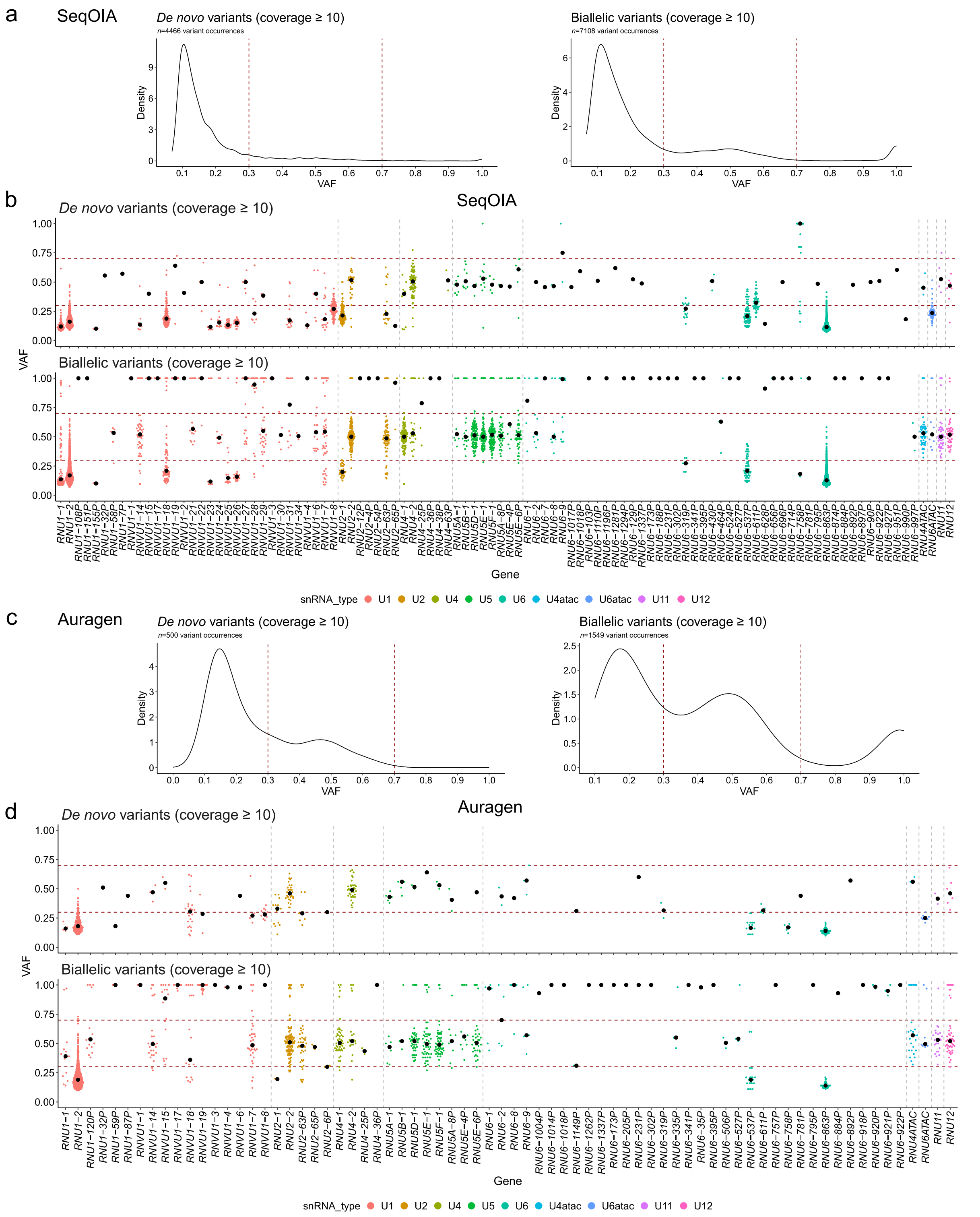

Supplementary Fig. 2. Variant allele fraction (VAF) distributions of snRNA variants in SeqOIA and Auragen subcohorts. a, VAF distribution of *de novo* (left panel) or biallelic (right panel) variants in snRNA genes with coverage ≥10 in SeqOIA. **b,** Detailed VAF distribution in SeqOIA. **c,** VAF distribution of *de novo* (left panel) or biallelic (right panel) variants in snRNA genes with coverage ≥10 in Auragen. **d,** Detailed VAF distribution in Auragen.

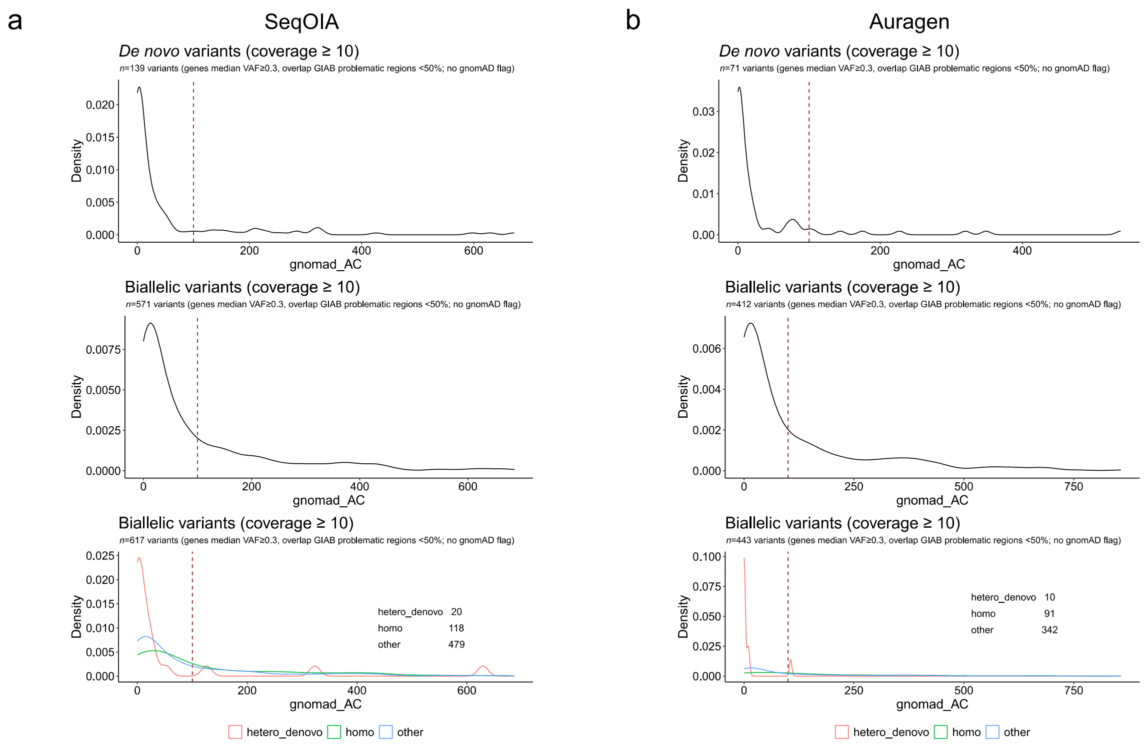

Supplementary Fig. 3. gnomAD allele count (AC) distribution of snRNA variants variants in SeqOIA and Auragen subcohorts. a, SeqOIA; **b,** Auragen. Upper panel: *de novo* variants with coverage ≥10. Middle and lower panels: biallelic variants with coverage ≥10 altogether (middle) or stratified according to their inheritance (lower).

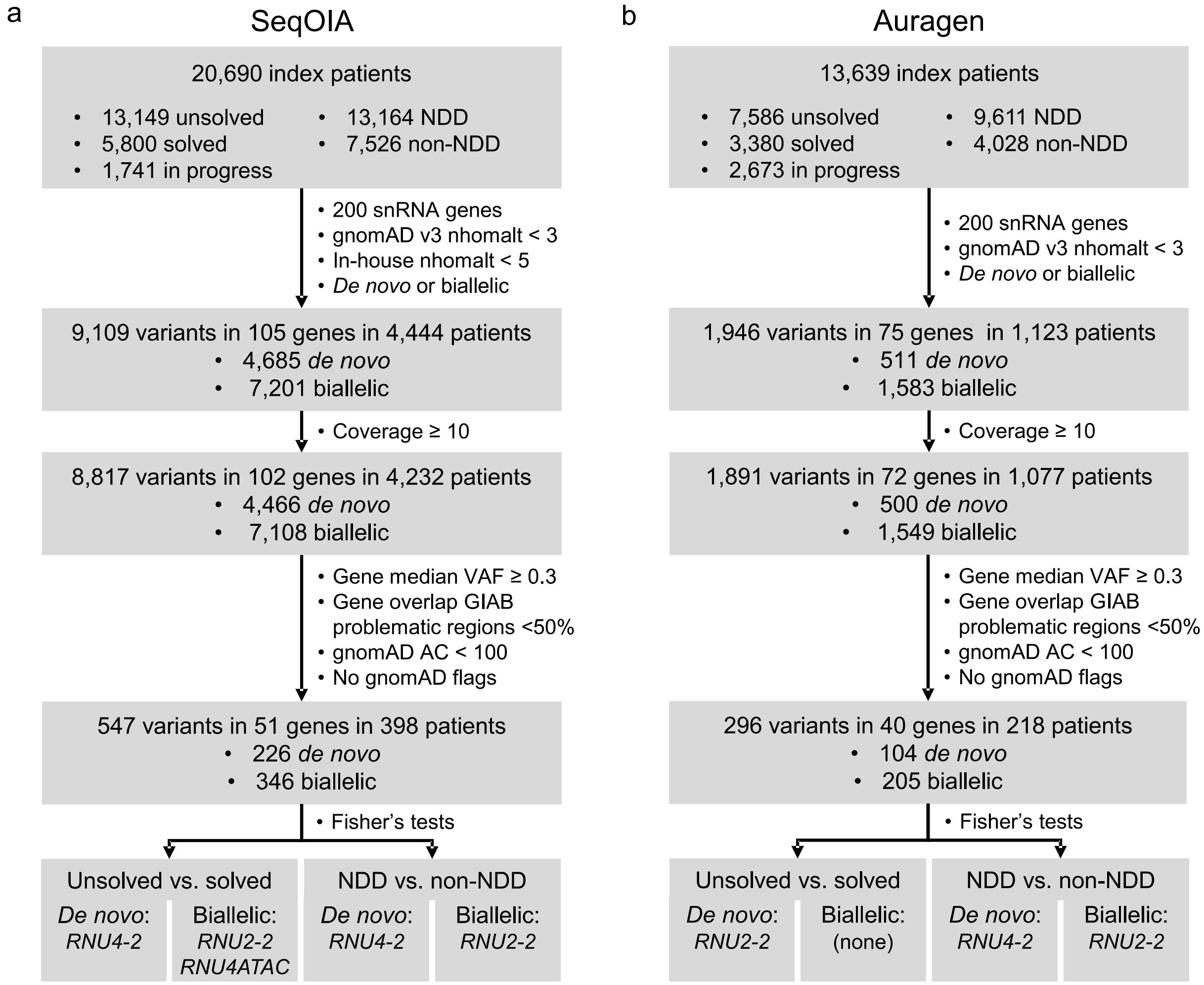

Supplementary Fig. 4. Details of the PFMG subcohorts and variant filtering strategy. Number of patients and variants in the entire PFMG cohort (**a**), or in the SeqOIA (**b**) and Auragen (**c**) subcohorts. Note that the variant numbers include multiple occurrences of the same variants.

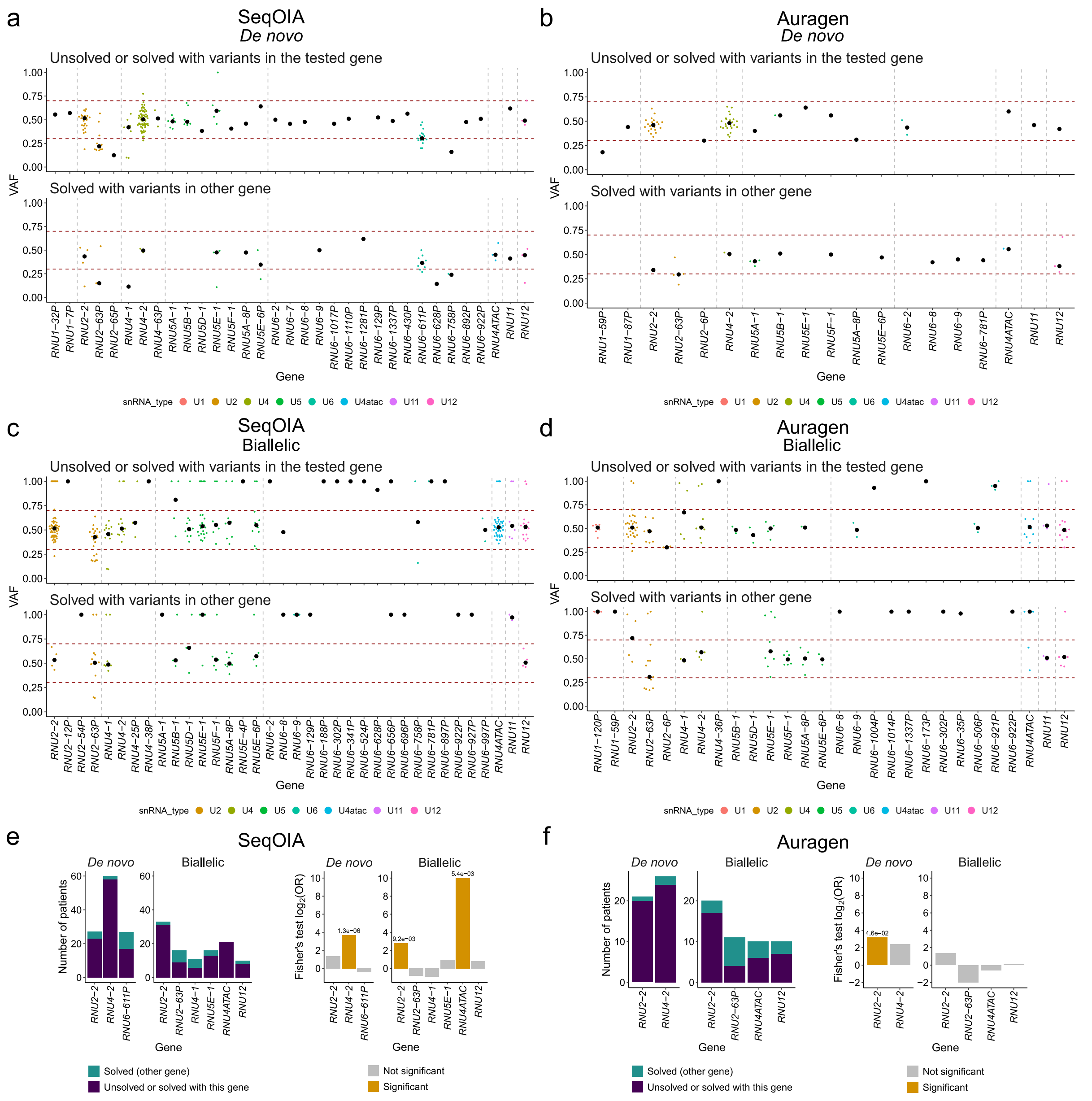

Supplementary Fig. 5. Comparison of rare variant burden in snRNA genes in unsolved versus solved cases separating the PFMG cohort into SeqOIA and Auragen subparts. a and **b,** Comparison of rare (gnomAD allele count < 100) *de novo* variants in SeqOIA (**a**) and Auragen (**b**). **c**, and **d,** Comparison of rare (gnomAD allele count < 100) biallelic variants in SeqOIA (**c**) and Auragen (**d**). **e** and **f,** Number of patients with rare *de novo* and biallelic variants (left panels) and statistical enrichment (two-sided Fisher's test) in unsolved versus unsolved cases (right panels) for genes in which at least 10 patients had variants (minimum number needed to reach statistical significance in the cohort) in SeqOIA (**e**) and Auragen (**f**). The number of patients per group and gene are shown in Supplementary Table 3.

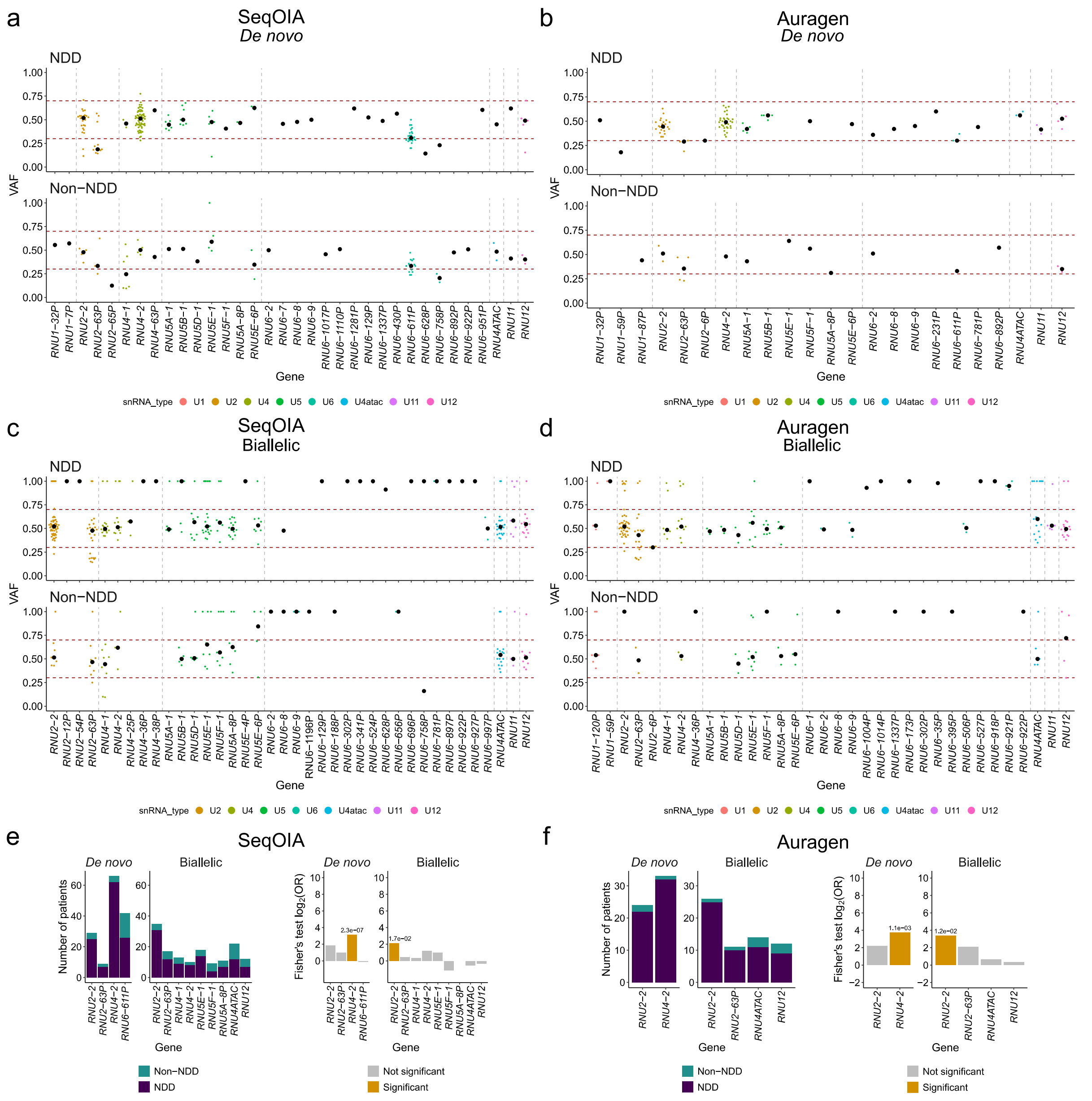

Supplementary Fig. 6. Comparison of rare variant burden in snRNA genes in NDD versus non-NDD cases separating the PFMG cohort into SeqOIA and Auragen subparts. a, and **b,** Comparison of rare (gnomAD allele count < 100) *de novo* variants in SeqOIA (**a**) and Auragen (**b**); **c**, and **d,** Comparison of rare (gnomAD allele count < 100) biallelic variants in SeqOIA (**c**) and Auragen (**d**). **e,** and **f,** Number of patients with rare *de novo* and biallelic variants (left panels) and statistical enrichment (two-sided Fisher's test) in NDD versus non-NDD cases (right panels) for genes in which at least 9 (SeqOIA; **e**) or 10 (Auragen; **f**) patients had variants (minimum number needed to reach statistical significance in the cohort). The number of patients per group and gene are shown in Supplementary Table 5.

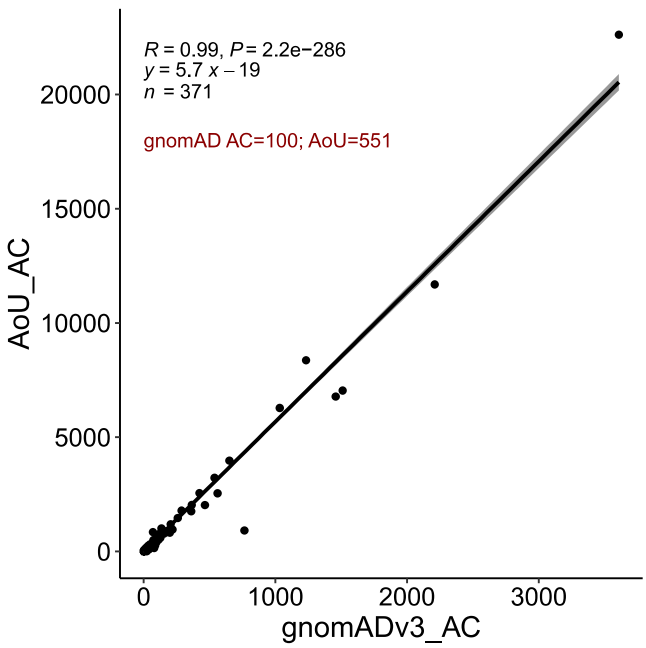

Supplementary Fig. 7. Pearson's correlation between gnomAD v3 and All of Us allele counts for *RNU2-2* variants. The cut-off of 100 allele counts (AC) in gnomAD v3.1 (76,156 genomes) corresponds to 551 in the *All of Us* database (414,000 genomes). For the PFMG cohort, we applied stricter thresholds: AC<50 for *de novo* variants and AC<200 for biallelic variants, representing 10× and 2.5× greater stringency, respectively, compared to the gnomAD cut-off.
