# Supplementary material for: Systematic analysis of snRNA genes reveals frequent RNU2-2 variants in dominant and recessive developmental and epileptic encephalopathies
Source: Nat Genet. 2026 Mar 30;58(4):782–97. doi: 10.1038/s41588-026-02547-5 (PMC13083260; doi:10.1038/s41588-026-02547-5)
Supplement: Supplementary file 2 — Reporting Summary [file 41588_2026_2547_MOESM2_ESM.pdf]

nature portfolio

Corresponding author(s): Christel Depienne, Gaetan Lesca and Caroline Nava

Last updated by author(s): Feb 2, 2026

Reporting Summary

Nature Portfolio wishes to improve the reproducibility of the work that we publish. This form provides structure for consistency and transparency in reporting. For further information on Nature Portfolio policies, see our Editorial Policies and the Editorial Policy Checklist.

Statistics

For all statistical analyses, confirm that the following items are present in the figure legend, table legend, main text, or Methods section.

n/a	Confirmed	
☐	☒	The exact sample size (n) for each experimental group/condition, given as a discrete number and unit of measurement
☒	☐	A statement on whether measurements were taken from distinct samples or whether the same sample was measured repeatedly
☐	☒	The statistical test(s) used AND whether they are one- or two-sided<br>*Only common tests should be described solely by name; describe more complex techniques in the Methods section.*
☐	☒	A description of all covariates tested
☐	☒	A description of any assumptions or corrections, such as tests of normality and adjustment for multiple comparisons
☐	☒	A full description of the statistical parameters including central tendency (e.g. means) or other basic estimates (e.g. regression coefficient) AND variation (e.g. standard deviation) or associated estimates of uncertainty (e.g. confidence intervals)
☐	☒	For null hypothesis testing, the test statistic (e.g. F, t, r) with confidence intervals, effect sizes, degrees of freedom and P value noted<br>*Give P values as exact values whenever suitable.*
☒	☐	For Bayesian analysis, information on the choice of priors and Markov chain Monte Carlo settings
☒	☐	For hierarchical and complex designs, identification of the appropriate level for tests and full reporting of outcomes
☐	☒	Estimates of effect sizes (e.g. Cohen's d, Pearson's r), indicating how they were calculated

Our web collection on statistics for biologists contains articles on many of the points above.

Software and code

Policy information about availability of computer code

Data collection	Variants in genes encoding snRNA in the PFMG were accessed using custom scripts. gLEAVES, the system used for genome analysis on the SeqIOA laboratory which is restricted to registered and accredited users, was used to investigate inheritance of variants and visualize bam files. Custom scripts were used on the Auragen. Data from additional cohorts were collected though national collaborative networks in France, or established collaborations with the Broad Institute, the Mefford lab, the Gleeson lab and the EPICARE ERN. We additionally used data from the publicly available gnomAD v3.1.2 (https://gnomad.broadinstitute.org/), and All of Us (https://databrowser.researchallofus.org/) databases.
Data analysis	The following software and analysis tool were used: Geneious Prime® 2019.2.3, Seqscape v2.6, STAR aligner v.2.7.11a, CIBERSORTx v1.0, rMATS v.4.3.0, rmats2sashimi, statsmodels v.0.14.5, seaborn v0.13.2, Meffil R package, ggplot2 v3.3.6, pheatmap v1.0.12, stats v4.2.0, and PyMol v3.0.0. Variants were reviewed using MobiDetails (https://mobidetails.iurc.montp.inserm.fr/MD/). Bam files were visualized with IGV 2.19.7. We also used data from Ensembl Release 112 and data from ENCODE Consortium: bigwig files with the plus/minus strand signals of unique reads from the default anisogenic replicate from the following tissues: diencephalon (https://www.encodeproject.org/experiments/ENCSR000AFR/), parietal lobe (https://www.encodeproject.org/experiments/ENCSR000AFY/), occipital lobe, (https://www.encodeproject.org/experiments/ENCSR000AFX/), frontal cortex (https://www.encodeproject.org/experiments/ENCSR000AFS/), temporal lobe (https://www.encodeproject.org/experiments/ENCSR000AGD/), cerebellum (https://www.encodeproject.org/experiments/ENCSR000AFQ/).

For manuscripts utilizing custom algorithms or software that are central to the research but not yet described in published literature, software must be made available to editors and reviewers. We strongly encourage code deposition in a community repository (e.g. GitHub). See the Nature Portfolio guidelines for submitting code & software for further information.

Data

Policy information about availability of data

All manuscripts must include a data availability statement. This statement should provide the following information, where applicable:

- Accession codes, unique identifiers, or web links for publicly available datasets
- A description of any restrictions on data availability
- For clinical datasets or third party data, please ensure that the statement adheres to our policy

Variant details have been submitted to ClinVar (SUB15855213; https://www.ncbi.nlm.nih.gov/clinvar/?term=SUB15855213). RNA-seq and methylation data have been deposited in the European Genome-phenome Archive (EGA, http://www.ebi.ac.uk/ega), hosted by the EBI. RNAseq data are available under the Study Accession Number EGAS50000001410 (https://ega-archive.org/studies/EGAS50000001410). Methylation data are accessible under the study accession EGAS00001008070 (https://ega-archive.org/studies/EGAS00001008070). Both are subject to a Data Processing Agreement, and access requests will be reviewed by a Data Access Committee to ensure compliance with ethical and legal standards. Due to ethical considerations, individual genome data cannot be made publicly available. Controlled access is required to safeguard participant privacy and to comply with data protection regulations, including the GDPR in Europe. Access to genome data from the PFMG2025 cohort is governed by French data protection laws and is only possible via the Collecteur Analyseur de Données (CAD). More details can be found on the PFMG2025 website: https://pfmg2025.fr/le-plan/collecteur-analyseur-de-donnees-cad/. The coordinates of the ENCODE Registry of candidate cis-Regulatory Elements (cCREs) in the human genome26 and bigwig files concerning the peaks of histone H3 acetylation of lysine 27 (H3K27Ac) obtained for the H1 human embryonic stem cell line (H1-hESC)27 were downloaded through the UCSC Table Browser60. Bigwig files concerning small RNA-seq data from six human embryonic brain regions were downloaded from the ENCODE portal61 (https://www.encodeproject.org/) with the following identifiers: ENCFF013RLG, ENCFF029RIV, ENCFF034QAV, ENCFF197SSE, ENCFF221KEN, ENCFF343ZBS, ENCFF405QIN, ENCFF532SOY, ENCFF654ONK, ENCFF738LDD, ENCFF870FMA, ENCFF887TOS, ENCFF106ESQ, ENCFF222WBQ, ENCFF250WEA, ENCFF254UEQ, ENCFF319GRF, ENCFF425WUZ, ENCFF443ONL, ENCFF820JTT, ENCFF897IWP, ENCFF915WAC, ENCFF946YVE and ENCFF965GHD.

Research involving human participants, their data, or biological material

Policy information about studies with human participants or human data. See also policy information about sex, gender (identity/presentation), and sexual orientation and race, ethnicity and racism.

Reporting on sex and gender	We obtained information on patients' sex from clinical records and referring physicians. Data on gender were not collected.
Reporting on race, ethnicity, or other socially relevant groupings	We collected information on the geographic origin and reported ancestry of a subset of participants; however, these data were not used in any analyses.
Population characteristics	Our study report RNU2-2 variants in 141 patients from 122 unrelated families (88 from PFMG cohort and 34 from other cohorts). We collected detailed clinical data for 112 of them (55 females and 57 males). The median age at inclusion in the study was 13 years (range: 0 (fetus) - 46 years).
Recruitment	The main cohort is composed of patients with rare disorders and their parents when available, who underwent genome sequencing as part of the diagnostic process in France (Plan France Médecine Génomique 2025, PFMG2025).At the time of the study, this cohort comprised short-read genome data from 34,329 patients with rare disorders Analysis of 200 snRNA genes lead to include 42 participants with rare de novo variants in one of these genes and 60 individuals from 52 families with biallelic variants in RNU2-2. Furthermore, date of 34 additional patients from other cohorts (13 with de novo variants and 21 with biallelic variants) were collected through international collaborations.
Ethics oversight	This study was conducted in accordance with the ethical standards and regulations of all participating countries. Written informed consent was obtained for all patients from their parents or legal guardians, with an additional consent form for families agreeing to the publication of photographs. For genetic analyses, patient samples were pseudonymized at each participating center. Information on the patients' sex (but not gender) was extracted from clinical records. The promoters of this research study are Assistance Publique–Hôpitaux de Paris (AP-HP) for hospitals associated with the SeqOIA laboratory (project ID APHP241333) and Grenoble-Alpes University Hospital (CHU Grenoble-Alpes, research ID 19814188) for hospitals affiliated with the Auragen laboratory. Ethical approval was obtained from the University Hospital Essen (24-12010-BO) and the Comité Éthique et Scientifique pour les Recherches, les Études et les Évaluations dans le domaine de la Santé (CESREES; reference 21082803 Bis / 2038764). AP-HP has received an authorization from the Commission Nationale de l'Informatique et des Libertés (CNIL; reference HGTHGT/MFIMFI/AR2426865; request no. 924924336666) for data processing. Additional approvals were obtained from the ethics committee of CHU de Nantes (CCTIRS number 14.556) and from CPP Ouest V (File 06/15, Ref MESR DC 2017 2987; approval date 04/08/2015). For methylation analyses, DNA from patients and controls had been previously collected in a medical context for genetic testing, with written consent including authorization for research use of leftover material. Control samples consisted of individuals without neurodevelopmental disorders, either unaffected relatives or persons tested presymptomatically for other conditions who were found not to carry pathogenic variants. DNA samples used for methylation profiling were stored within the genetics biobank of the CRBi, Rouen, France (collection DC 2008-711, authorization MCRBi/2024/02). The use of these samples was approved by the CERDE ethics committee of Rouen University Hospital (notification E2023-13). Researchers and clinicians from all contributing centers participated throughout the study, from design and implementation to data collection, analysis and manuscript preparation, and are listed as coauthors of this article.

Note that full information on the approval of the study protocol must also be provided in the manuscript.

Field-specific reporting

Please select the one below that is the best fit for your research. If you are not sure, read the appropriate sections before making your selection.

☒ Life sciences ☐ Behavioural & social sciences ☐ Ecological, evolutionary & environmental sciences

For a reference copy of the document with all sections, see nature.com/documents/nr-reporting-summary-flat.pdf

Life sciences study design

All studies must disclose on these points even when the disclosure is negative.

Sample size	The main cohort (PFMG) comprised 34,329 patients with rare disorders, including 22,775 individuals with NDD (and their parents when available). These numbers represent all genomes included in the PFMG2025 cohort at the time of study closure (May 2025 for Auragen and November 2025 for SeqOIA). No formal sample size calculation was performed, as this was an exploratory study not designed to test a predefined hypothesis. The same rationale applied to RNA-seq and DNA methylation analyses: biological material (blood samples and extracted DNA) was collected from all families who consented to participate or for whom suitable material was already available.
Data exclusions	BAM files were visualized in IGV, and only confirmed de novo variants were included in further analysis. Variants resulting from mismapped reads (regions with multiple variants) or 'de novo' variants also detectable in reads of the parents after inspection of Bam files were discarded.
Replication	We replicated the main findings of this study by collecting data from 34 additional patients with de novo or biallelic variants in RNU2-2 from independent cohorts. Moreover, the key results have been independently reproduced by two separate groups (Jackson et al., medRxiv; Greene et al., medRxiv). No findings reported in this study failed independent replication.
Randomization	Not applicable (observational study)
Blinding	Not applicable (observational study)

Reporting for specific materials, systems and methods

We require information from authors about some types of materials, experimental systems and methods used in many studies. Here, indicate whether each material, system or method listed is relevant to your study. If you are not sure if a list item applies to your research, read the appropriate section before selecting a response.

Materials & experimental systems

n/a	Involved in the study	
☒	☐	Antibodies
☒	☐	Eukaryotic cell lines
☒	☐	Palaeontology and archaeology
☒	☐	Animals and other organisms
☒	☐	Clinical data
☒	☐	Dual use research of concern
☒	☐	Plants

Methods

n/a	Involved in the study	
☒	☐	ChIP-seq
☒	☐	Flow cytometry
☒	☐	MRI-based neuroimaging

Plants

Seed stocks	na
Novel plant genotypes	na
Authentication	na
